# Transcriptome Profiling of Osteoblasts in a Medaka (*Oryzias latipes*) Osteoporosis Model Identifies Mmp13b as Crucial for Osteoclast Activation

**DOI:** 10.3389/fcell.2022.775512

**Published:** 2022-02-21

**Authors:** Ranran Liu, Nurgul Imangali, Lalith Prabha Ethiraj, Tom James Carney, Christoph Winkler

**Affiliations:** ^1^ Department of Biological Sciences and Centre for Bioimaging Sciences, National University of Singapore, Singapore, Singapore; ^2^ Lee Kong Chian School of Medicine, Nanyang Technological University, Singapore, Singapore

**Keywords:** osteoblasts, osteoclasts, matrix metalloprotease, medaka (*Oryzias lapites*), osteoporosis

## Abstract

Matrix metalloproteases (MMPs) play crucial roles in extracellular matrix (ECM) modulation during osteoclast-driven bone remodeling. In the present study, we used transcriptome profiling of bone cells in a medaka model for osteoporosis and bone regeneration to identify factors critical for bone remodeling and homeostasis. This identified *mmp13b*, which was strongly expressed in osteoblast progenitors and upregulated under osteoporotic conditions and during regeneration of bony fin rays. To characterize the role of *mmp13b* in bone remodeling, we generated medaka *mmp13b* mutants by CRISPR/Cas9. We found that *mmp13b* mutants form normal numbers of osteoblasts and osteoclasts. However, osteoclast activity was severely impaired under osteoporotic conditions. In *mmp13b* mutants and embryos treated with the MMP13 inhibitor CL-82198, unmineralized collagens and mineralized bone matrix failed to be degraded. In addition, the dynamic migratory behavior of activated osteoclasts was severely affected in *mmp13b* mutants. Expression analysis showed that maturation genes were downregulated in *mmp13b* deficient osteoclasts suggesting that they remain in an immature and non-activated state. We also found that fin regeneration was delayed in *mmp13b* mutants with a concomitant alteration of the ECM and reduced numbers of osteoblast progenitors in regenerating joint regions. Together, our findings suggest that osteoblast-derived Mmp13b alters the bone ECM to allow the maturation and activation of osteoclasts during bone remodeling in a paracrine manner. Mmp13b-induced ECM alterations are also required to facilitate osteoblast progenitor recruitment and full regeneration of bony fin rays.

## Introduction

A combination of controlled resorption of old bone matrix by osteoclasts and formation of new matrix by osteoblasts at sites of bone remodeling is needed to maintain a healthy skeleton (reviewed by Hadjidakis and Androulakis, 2006; reviewed by Raggatt and Partridge, 2010). The respective cell activities are coordinated by known signaling pathways. For osteoclasts, signaling by M-CSF/c-Fms and RANKL-RANK pathways has been shown to induce osteoclast differentiation and bone resorption (reviewed by Boyle et al., 2003). Osteoblast differentiation on the other hand depends on signaling most prominently through the WNT and BMP pathways (reviewed by Fakhry et al., 2013). Besides growth factor signaling, contact-dependent cell-cell and cell-extracellular matrix (ECM) interactions are also required for bone cell activation. For instance, the resorptive activity of mature osteoclasts is controlled by direct cell-cell contact with osteoblasts (Furuya et al., 2018). Osteoclasts also require close contact to ECM to initiate bone resorption, which involves binding of integrins such as αVβ3 on the osteoclast surface to RGD peptides made available in the ECM (reviewed by Alford et al., 2015). In many cases, these interactions are triggered by the activity of matrix metalloproteases (MMPs), which are extracellular, zinc-dependent endopeptidases that degrade bone matrix proteins, such as collagens, and modulate the structure and composition of ECM (reviewed in Hardy and Fernandez-Patron, 2020). MMPs are expressed by osteoblasts, osteocytes and osteoclasts and exhibit various functions in controlling bone cell behavior during bone remodeling (reviewed by Paiva and Granjeiro, 2017). Several MMPs were earlier described to be secreted into bone resorption lacunae, including MMP2, MMP9, MMP13, and MMP14 (reviewed by Paiva and Granjeiro, 2017). Interestingly, in this process, non-osteoclastic cells such as osteoblasts produce MMP2 and MMP13, while osteoclasts express MMP9 and MMP14. The fact that a combination of both osteoblast- and osteoclast-derived MMPs contributes to resorptive activity implies a possible role of MMPs in bone cell coupling to control osteoclast activity (Yamagiwa et al., 1999; Andersen et al., 2004; Zhu et al., 2020). When the intricate communication between osteoclasts and osteoblasts and their respective interactions with ECM components is impaired, it leads to excessive bone resorption and osteoporosis (reviewed in Feng and McDonald, 2011). Interestingly, MMPs are also implicated in bone formation. Accordingly, in *Mmp2−/−* mice bone mineralization was impaired and numbers of both osteoblasts and osteoclasts reduced (Mosig et al., 2007). This indicates a complex interplay of distinct MMPs produced by different bone cell types during bone remodeling. It, however, remains largely unclear, how osteoblasts, whose main role is to deposit bone matrix, contribute to the activation of osteoclasts by modulating the ECM.

In teleost fish, osteoblasts are key drivers for bone formation in the regenerating fins. This process involves dedifferentiation as well as *de novo* formation of osteoblasts, which produce the ECM template needed for remineralization of the regenerating fin rays (Knopf et al., 2011; reviewed by Sehring and Weidinger, 2020; Singh et al., 2012). We recently identified two populations of *col10a1*-positive osteoblast progenitors that contribute to the formation of segmental osteoblasts and new joint cells in the regenerating fins (Dasyani et al., 2019).

To identify common and distinct molecular aspects of osteoblast-driven bone remodeling during osteoporosis and bone regeneration, we utilized a medaka model, in which excessive bone resorption can be induced by transgenic Rankl expression (To et al., 2012; Phan et al., 2020b). We compared the transcriptome profiles of *col10a1* osteoblast progenitors in medaka embryos after Rankl induction and in adult caudal fins at 2 days post amputation (dpa). This identified a significant up-regulation of *mmp13b* under both conditions. Earlier studies on human breast tumors had shown that MMP13 promotes formation of osteoclasts to trigger bone resorption and bone metastasis (Pivetta et al., 2011). On the other hand, morpholino-mediated knock-down of *mmp13a* in zebrafish resulted in impaired differentiation of osteoblasts (Li et al., 2020). In medaka, other than in zebrafish, bone is acellular and lacks osteocytes [(Ofer et al., 2019); PMID: 30707688]. Osteocytes are terminally differentiated osteoblasts that are embedded in bone matrix and produce MMP13 (Jáuregui et al., 2016). In this cellular context, MMP13 is needed for dynamic remodeling of bone matrix in lacunar-canalicular networks that osteocytes use to communicate with other osteocytes, osteoblasts and osteoclasts to regulate bone density (reviewed in Moharrer and Boerckel, 2021). Together, this suggests distinct roles of Mmp13 in complex interplays of various bone cell types to control formation and activity of osteoblasts and osteoclasts that are not fully understood.

Here, we generated *mmp13b* knock-out medaka mutants and characterized its function in bone remodeling and fin regeneration. We show that *mmp13b* is strongly expressed in osteoblast progenitors and that enzymatic metalloprotease activity is elevated in osteoblasts after Rankl induction. We further demonstrate that Mmp13b promotes osteoclast maturation and activity under osteoporotic conditions. Excessive bone formation in *mmp9* ^*−/−*^;*mmp13* ^*−/−*^ double-mutants without Rankl induction suggests that also the activity of endogenous osteoclasts is impaired. In addition, *mmp13b* mutants exhibit delayed fin regeneration and altered ECM composition with reduced numbers of osteoblast progenitors in joints. Our study therefore shows that osteoblast derived Mmp13 is necessary for progenitor recruitment and for osteoclast activation in a non-cell autonomous fashion.

## Materials and Methods

### Maintenance of Medaka Lines

Transgenic medaka were maintained in the fish facility at the Department of Biological Sciences, National University of Singapore (DBS, NUS). All breeding and experimental procedures were conducted under protocols approved by the Institute of Animal Care and Use Committee of the National University of Singapore (IACUC; protocol numbers: BR19-0120, R18-0562).

### Isolation of Bone Cells and RNA Sequencing

Purification of medaka bone cells by fluorescence activated cell sorting (FACS) and RNA sequencing (RNAseq) was described previously (Buettner et al., 2018; Phan et al., 2020b). Briefly, *rankl*:HS:*cfp* transgenic larvae with *col10a1*:GFP, *osx*:mCherry, or *ctsk*:GFP reporter backgrounds were heat-shocked at 9 days post fertilization (dpf) at 39°C for 2 h to collect *col10a1*-positive osteoblast progenitors and *osx*-positive osteoblasts at 10 dpf, and *ctsk*-positive osteoclasts at 12 dpf. Transgenic reporter larvae without Rankl transgene were used as control. *mmp13b* ^+/+^ and *mmp13b* ^−/−^ larvae with *rankl*:HS:*cfp*/*ctsk*:mCherry transgenic background were heat-shocked at 9 dpf at 39°C for 2 h and used to collect mCherry-positive osteoclasts at 3 days post heat-shock (dphs). To collect GFP-positive osteoblast progenitors from caudal fins, 2–3 month-old *col10a1*:GFP transgenic fish were used. Tissue dissociation was performed using a Papain Dissociation System (Worthington Biochemical Corporation) following the manufacturer’s instructions. FACS was conducted using a BD FACS Aria II 5 lasers cytometer. RNA was isolated from FACS purified cells using the Purelink™ RNA micro kit (Invitrogen). RNA quality was measured with an Agilent Bioanalyzer 2100 and Agilent RNA 6000 Pico Kit (Agilent Technologies, United States), and samples with a RIN value > 8.0 were sent to Novogene for RNA sequencing and bioinformatics analysis. The sequencing results were deposited to NCBI’s BioProject, under accession number PRJNA767186.

### Quantitative RT-PCR

RNA was isolated from FACS purified cells and whole mount fins at 2 days post amputation (dpa) using a Purelink™ RNA micro kit (Invitrogen) and cDNA was synthesized and amplified using a PreAmp and Reverse Transcription Master Mix Kit (Fluidigm). qPCR was performed with PowerUp SYBR Green Master Mix (Applied Biosystems). All primers used for qPCR are listed in Supplementary Table S3. *β-actin* was used for normalization. All experiments were performed with three biological samples and three technical repeats each. Samples for RNAseq analysis and qPCR validation were obtained from independent experiments. Data were analyzed using the Bio-Rad CFX Maestro 1.0 software and Student’s t test was performed for statistical analysis.

### RNA *in situ* Hybridization

Riboprobes spanning 659 nucleotides of the *mmp13b* cDNA (nt458-nt1116; ENSORLG00000014453; http://www.ensembl.org/) were synthesized using a DIG RNA labeling kit (Roche, CAT# 11175025910). The synthesis of a *col10a1* riboprobe was described previously (Renn et al., 2013). RNA *in situ* hybridization was conducted on cryosections as described previously (McMillan et al., 2018) with small modifications. The NBT/BCIP (Sigma) staining solution was added onto the sections and stained at room temperature. Then, the sections were washed with PBS-Tween 20 (PBST) and mounted in Mowiol 4-88 (Calbiochem) for imaging.

### Generation of *mmp9* and *mmp13b* Mutants

Guide RNAs (gRNAs) targeting *mmp9* (ENSORLG00000006340) and *mmp13b* (ENSORLG00000014453) were designed using the CRISPR/Cas9 target online prediction tool (CCTop) (Stemmer et al., 2015) and synthesized by Integrated DNA Technologies (IDT Singapore; sequences in Supplementary Table S3). Three gRNAs (36 ng/μL each) were co-injected into one-cell stage medaka embryos together with tracrRNA (67 ng/μL, IDT) and Cas9 nuclease (0.25 μg/μL, IDT). Primers used for genotyping (*mmp9*_FP/RP, *mmp13b_*FP/RP) are listed in Supplementary Table S3.

### MMP13 Inhibitor Treatment

The selective MMP13 inhibitor CL-82198 hydrochloride (TOCRIS, CAT# 2632) was dissolved in H_2_O to make a 50 mM stock solution. The stock solution was stored at room temperature and working solutions were prepared freshly for every use. Pre-treatment was performed on *rankl*:HS:*cfp* transgenic larvae from 7–9 dpf with 300 μM CL-82198 (12 μl stock in 2 ml fish medium). Larvae were heat-shocked at 9 dpf to induce Rankl expression, followed by consecutive treatment from 9–12 dpf with 300 μM CL-82198. Control fish were treated with 12 μl H_2_O in 2 ml fish medium from 7–12 dpf. All larvae were kept at 30°C in an incubator throughout the treatment period.

### Bone Staining

12 dpf larvae were transferred to 0.004% calcein staining solution for 1 hour at 30°C in the dark. Then, the larvae were washed with fish medium before mounting in 1.2% low melting agarose for imaging. Alizarin Red bone staining of 12 dpf larvae and 17 dpa fins was performed as previously described (Renn and Winkler, 2009).

### Quantification of Osteoclast Numbers and Lesion Sizes

Fluorescent images of whole embryos were used for the quantification of osteoclast numbers following the method described previously (Phan et al., 2020a). For quantification of bone lesion sizes, four consecutive vertebral bodies were used for each analysis. The lesion percentage was determined as 100%*[0.5* (lesion area/total area) + 0.5* (number of absorbed arches/4)]. For statistical analysis, a Student’s t test (two-tailed, unpaired) was performed using GraphPad Prism 6.0 (GraphPad Software, La Jolla, CA, United States).

### Picro Sirius Red and Immunostaining

Collagen staining was performed on 20 μm cryosections using a Picro Sirius Red Stain Kit (ab150681; Abcam, UnitedStates) following the manufacturer’s instructions. Cryosection slides were defrosted at room temperature for 5 min before rehydration in water. Sections were incubated with Picro sirius red staining solution for 1 h and thereafter rinsed with acetic acid before dehydration with absolute ethanol and mounting in synthetic resin for imaging.

Immunostaining on 20 μm cryosections was performed as described previously (Phan et al., 2020a). The following primary antibodies were used: Rabbit anti-collagen 1 (ab23730, Abcam), mouse anti-collagen 2 (II-II6B3; Developmental Studies Hybrydoma Bank, United States), rabbit anti-fibronectin (F3648, Sigma, United States), rabbit anti-Tenascin (T2550-23, United States Biological, United States). As secondary antibodies, goat anti-rabbit Alexa Fluor 633 (Thermofisher Scientific) and goat anti-mouse Alexa Fluor 633 (Thermofisher Scientific) were used. The numbers of analyzed sections is listed in Supplementary Table S4.

### Tartrate Resistant Acidic Phosphatase Staining

TRAP staining on 20 μm cryosections was performed as described previously (Ethiraj et al., 2021). This included defrosting slides at room temperature, washing with PBS and incubating the slides with TRAP staining solution for 2 h. After this, slides were washed with PBS and mounted in Mowiol 4-88 for imaging.

Caudal fins were collected from 3 month-old adult medaka with *mpeg1*:mCherry transgenic background at 2 dpa and cryosectioned to obtain 20 μm longitudinal sections. Sections were stained with TRAP staining reagent for 6 h at room temperature. The same fin sections were imaged before and after TRAP staining.

### DQ Collagen *in situ* Zymography

Collagen *in situ* zymography was conducted as described before but with slight modifications (Bai et al., 2005; Li et al., 2020). Rankl-transgenic larvae were heat-shocked at 9 dpf and fixed at 12 dpf with 4% PFA at 4°C overnight. 20 μm transverse sections from the trunk were obtained by cryosectioning and dried at room temperature for 2 h, followed by PBST washing and counterstaining with DAPI. DQ collagen substrate conjugated with FITC (ThermoFisher Scientific; D12060) was utilized as substrate for *in situ* zymography. DQ collagen was dissolved in milliQ water to obtain a 1 mg/ml stock solution. 1% low-melting agarose (LMA) was dissolved in substrate buffer (50 mM Tris-HCl, 5.0 mM CaCl_2_, 5.0 μM ZnSO_4_; Ph 7.5). 200 μl gel containing 40 μl collagen stock solution, 60 μl substrate buffer, and 100 μl 1% LMA was coated on sections and sections were incubated at 37°C for 18 h. After incubation, sections were washed with PBST and mounted in Mowiol 4-88 for imaging. For negative control, slides were incubated at −20°C for 18 h.

### Imaging

For live fluorescence imaging, larvae were anesthetized with 0.016% Tricaine (MS222, Sigma), mounted in 1.2% low melting agarose on a glass-bottom Petri dish, and imaged using an Olympus FluoView FV3000 confocal microscope and a Nikon SMZ18 stereomicroscope equipped with the NIS-Elements BR 3.0 software. Olympus FluoView FV3000 confocal microscope was also used for time-lapse imaging and imaging of RNA *in situ* hybridization sections. The Nikon SMZ18 stereomicroscope was used to image Alizarin Red stained larvae and fins. To image Picro sirius red stained and TRAP-stained sections as well as RNA *in situ* hybridization sections, a Nikon Eclipse 90i upright microscope equipped with NIS-Elements BR 3.0 software was used. Immunostained sections were imaged with a LSM 900 Airyscan II super-resolution confocal laser scanning microscope. Images and time-lapse movies were processed with Bitplane Imaris and Fiji.

## Results

### *mmp13b* is Expressed in Medaka Osteoblast Progenitors and Upregulated Under Osteoporotic Conditions and in Regenerating Fins

**FIGURE 1:**
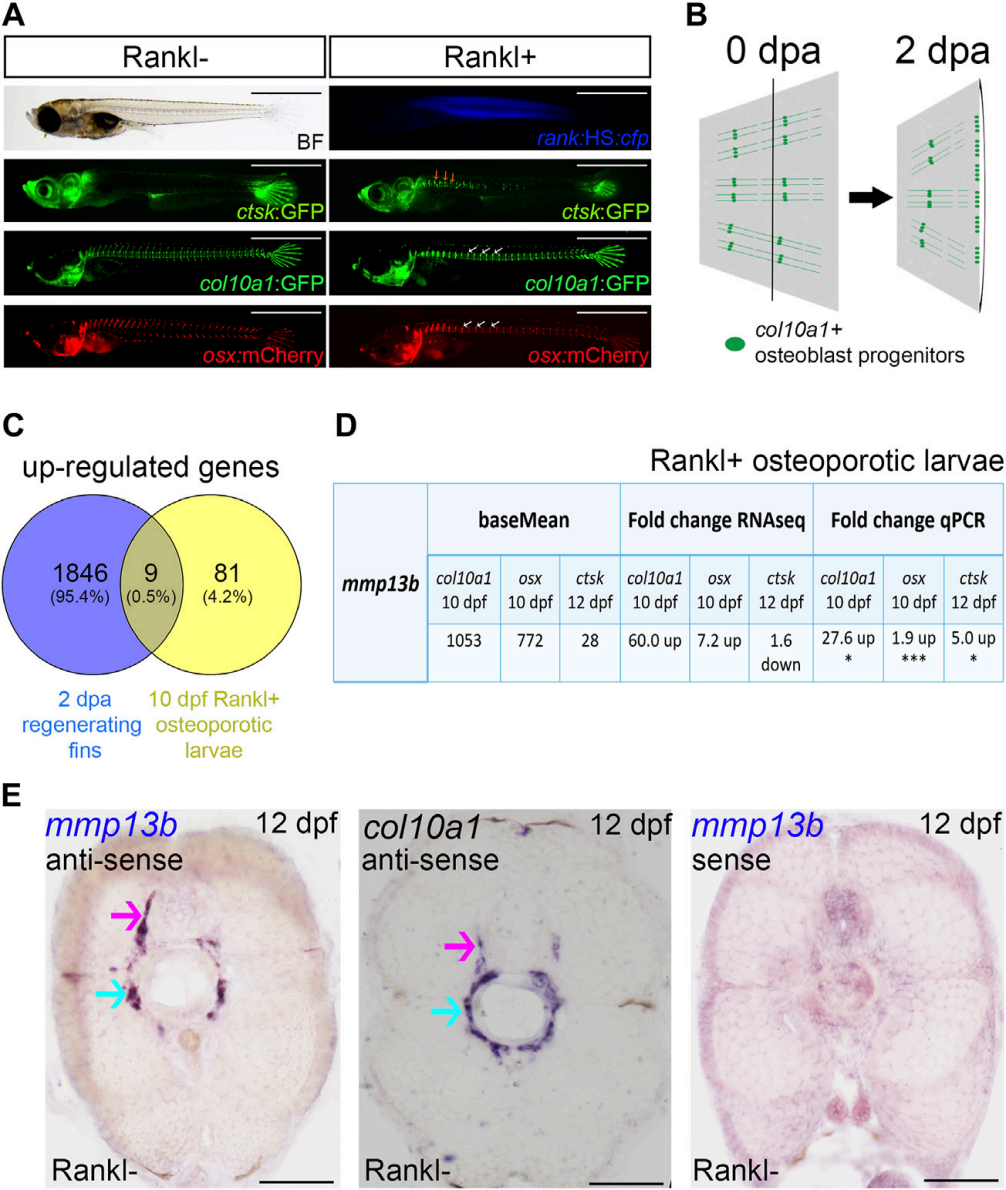
*mmp13b* is upregulated in bone remodeling and fin regeneration in medaka. **(A)** Medaka Rankl-induced osteoporosis model (for details, see Phan et al., 2020b; To et al., 2012). Transgenic lines used to visualize osteoclasts (*ctsk*:GFP), osteoblast progenitors (*col10a1*:GFP), and osteoblasts (*osx*:mCherry), respectively. Rankl induction is indicated by CFP expression. Ectopic osteoclast formation along the trunk (yellow arrows) after Rankl induction, followed by accumulation of osteoblast progenitors and osteoblasts in centra (white arrows). Scale bar: 1 mm. **(B)** Medaka fin regeneration. *col10a1*-positive osteoblast progenitors (green line and dots) are located along segments and in joints; after amputation, cells in joints migrate to the amputation plane (black line) for blastema formation and regeneration at 2 dpa. **(C)** Venn diagram showing total number of up-regulated genes expressed in osteoblast progenitors in 2 dpa regenerating fins and 10 dpf Rankl+ osteoporotic larvae, with the overlapping region showing commonly expressed genes. All genes have adjusted *p* values <0.05. **(D)** Table summarizing *mmp13b* baseMean and fold change values obtained by RNAseq and qPCR analysis, respectively. *mmp13b* is up-regulated in osteoblast progenitors (*col10a1*), osteoblasts (*osx*) at 10 dpf and osteoclasts (*ctsk*) at 12 dpf after Rankl induction. Student’s two-tailed *t* test. *p < 0.05, ***p < 0.001. **(E)** Endogenous *mmp13b* expression revealed by RNA *in situ* hybridization in 12 dpf medaka trunk cross-sections. *mmp13b* expression is found in the neural arch (magenta arrow) and the outer notochord surface (cyan arrow), where also *col10a1* is expressed. A *mmp13b* sense probe shows no staining. Scale bar: 50 μm.

To identify genes implicated in bone remodeling, we previously performed transcriptome profiling of bone cells in a medaka osteoporosis model where transgenic Rankl expression is induced by heat shock (Phan et al., 2020b; To et al., 2012). Differentially expressed genes (DEGs) were identified in *col10a1* positive osteoblast progenitors and *osterix* (*osx*) positive osteoblasts at 1 day post heat-shock (1 dphs; i.e., 10 days post fertilization, dpf) and in *cathepsin K* (*ctsk*) positive osteoclasts at 3 dphs (Figure 1A) (Phan et al., 2020b). In the present study, we performed RNA sequencing of *col10a1* cells isolated from regenerating caudal fins. In the medaka fin, *col10a1* positive osteoblast progenitors are located along segments of the mineralized hemirays and in joints linking the segments. After amputation, *col10a1* cells migrate to the amputation site and contribute to the regenerating fin rays (Dasyani et al., 2019; illustrated in Figure 1B). Here, we analyzed the transcriptomes of *col10a1* cells purified from fins at 0- and 2-days post amputation (dpa; Supplementary Figure S1). 14,377 transcripts were detected in *col10a1* cells (Supplementary Figure S1A), and among them, 3591 were differentially regulated, with 1855 significantly up-regulated at 2 dpa (Supplementary Figure S1B). A KEGG analysis revealed enrichment of, e.g., cell cycle genes, indicative for enhanced cell division of osteoblast progenitors during regeneration (Dasyani et al., 2019) as well as ECM-receptor interactions, among others (Supplementary Figure S1C).

Next, to identify genes commonly regulated during bone remodeling under osteoporosis-like lesions and in bone regeneration, we compared the regeneration dataset with the previously obtained osteoporosis dataset (Phan et al., 2020b). This revealed nine genes that were commonly upregulated in *col10a1* cells (Figure 1C; Table 1). One of them was *mmp13b*, which showed high expression levels and a significant fold change in both datasets. Quantitative RT-PCR (qPCR) analysis of FACS purified *col10a1* cells validated *mmp13b* up-regulation consistent with the idea that this metalloprotease is implicated in both bone resorption and repair. We also noticed *mmp13b* up-regulation in Rankl-induced (Rankl+) *ctsk* osteoclasts at 12 dpf. However, the baseMean expression levels were considerably lower than in osteoblasts (Figure 1D). To confirm expression of *mmp13b* in osteoblast progenitors, RNA *in situ* hybridization of wildtype embryos at 12 dpf showed *mmp13b* expression along neural arches (Figure 1E, magenta arrow) and the outer surface of the notochord (Figure 1E, cyan arrow), where *col10a1* cells reside. Together, this suggests that *mmp13b* is expressed in osteoblast progenitors, and that its expression is significantly upregulated under Rankl+ and regeneration conditions.

**TABLE 1:** Commonly upregulated genes in *col10a1* osteoblast progenitors of Rankl-induced medaka embryos at 10 dpf and in regenerating fins at 2 dpa.

Gene name	baseMean in osteoporotic embryos (10 dpf)	Read count in fin regeneration (2 dpa)	log2 FC in osteoporotic embryos (10 dpf)	log2 FC in fin regeneration (2 dpa)
*igfbp5a*	248	292	6.7	1.2
*mmp13b*	1,053	2,948	6.6	2.2
*gpha*	12	487	4	3.1
*itgb8*	394	220	4	3
*adam8*	99	343	3.5	0.93
*cmklr1*	1299	3741	3.4	1.3
*mmp14*	3,320	7,190	2.4	2.6
*alpl*	1,161	1,746	2.3	1.2
*soul2*	305	153	2.3	1.1

### Reduced Bone Resorption Under *mmp13b* Deficient Conditions

**FIGURE 2:**
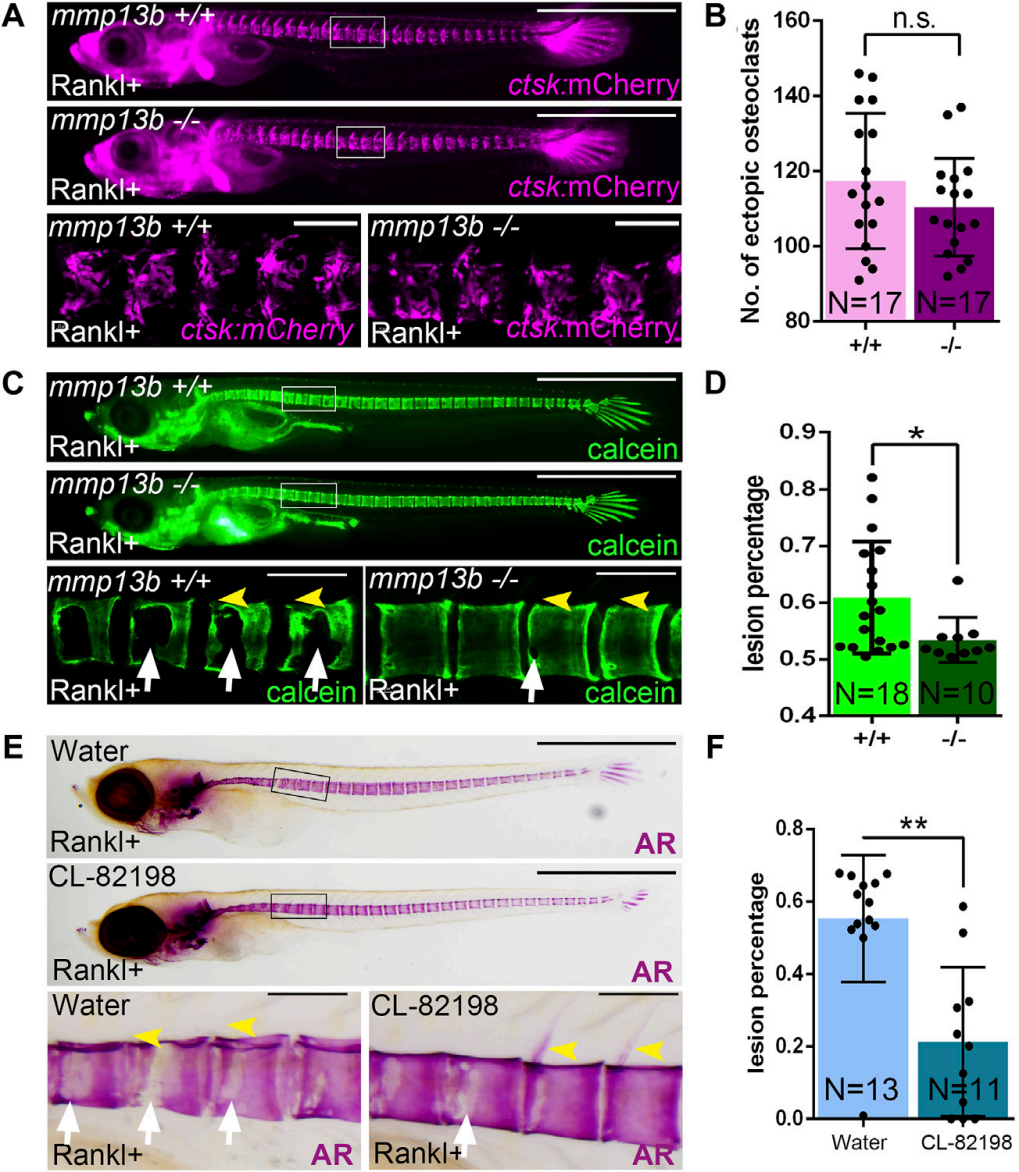
A *mmp13b* knock-out and chemical inhibition leads to reduced osteoclast resorption after Rankl induction. **(A)** Overview and high-magnification confocal images of *mmp13b* ^+/+^ and *mmp13b* ^−/−^ siblings in *rankl*:HS:*cfp*/*ctsk*:mCherry transgenic background at 2 dphs (11 dpf). Similar osteoclast formation in *mmp13b* ^+/+^ and *mmp13b* ^−/−^ at 2 dphs. **(B)** Quantification of *ctsk*:mCherry osteoclast numbers in vertebral column [region marked as white boxes in **(A)**] in *mmp13b* ^+/+^ and *mmp13b* ^−/−^ siblings at 2 dphs. **(C)** Overview and high-magnification confocal images of calcein stained mineralized matrix in *mmp13b* ^+/+^ and *mmp13b* ^−/−^ siblings at 3 dphs (12 dpf). Wildtype siblings but not homozygous mutants show severe resorption of mineralized matrix at neural arches (yellow arrowheads) and vertebral bodies (white arrows). **(D)** Quantification of bone lesions in vertebral column and neural arch [region marked as white boxes in **(C)**] in *mmp13b* ^+/+^ and *mmp13b* ^−/−^ siblings at 3 dphs. **(E)** Overview and high-magnification views of Alizarin red (AR) stained mineralized matrix in water and CL-82198 hydrochloride treated larvae at 3 dphs (12 dpf). Severe resorption of neural arches (yellow arrowheads) and vertebral bodies (white arrows) in control, while neural arches persisted and vertebral bodies had less resorption in treated larvae. **(F)** Quantification of bone lesions in vertebral column and neural arches [region marked as black boxes in **(E)**] in water and CL-82198 hydrochloride treated larvae at 3 dphs. Error bars indicate mean ± SD, *p < 0.05, **p < 0.01, ns, not significant, unpaired Student’s two-tailed *t* test. Scale bar: 1 mm (overview images), 100 μm (zoom in images).

We hypothesized that osteoblast-derived Mmp13b contributes to bone resorption in Rankl embryos and generated knock-out mutants using CRISPR/Cas9 to further characterize its function in medaka (Supplementary Figure S2). Homozygous *mmp13b* mutants were viable and showed no obvious phenotype during embryogenesis. After Rankl induction, ectopic osteoclasts formed normally at 2 dphs (11 dpf) with similar numbers in *mmp13b* ^+/+^ siblings and *mmp13b* ^−/−^ mutants (Figures 2A,B). Next, we assessed the extent of bone resorption after Rankl induction by calcein staining. In *mmp13b* ^*+/+*^ embryos, excessive bone resorption was evident, with lesions in the centra (Figure 2C; white arrows) and completely resorbed neural arches (yellow arrowheads). In contrast, *mmp13b* ^−/−^ mutants exhibited significantly less bone resorption with a 7.5% reduction in bone matrix lesions on average (Figure 2D). This suggests that a *mmp13b* deficiency does not affect formation of osteoclasts, but that Mmp13b is necessary for osteoclast activity.

To confirm this, we used the MMP13 inhibitor CL-82198 hydrochloride in medaka and examined bone resorption after Rankl induction. Like *mmp13b* mutants, CL-82198 treated embryos exhibited reduced bone resorption, when compared to controls, with less lesions in the vertebral centra (Figure 2E; white arrows) and mostly intact neural arches (yellow arrowheads). Quantifications revealed a significant reduction of the area with bone lesions in CL-82198 treated larvae compared to controls (Figure 2F), which was consistent with the phenotype in *mmp13b* knock-out mutants. Together, both knock-out of *mmp13b* and chemical inhibition with a MMP13 inhibitor in medaka led to impaired bone resorption after Rankl induction, suggesting a possible non-cell autonomous role in controlling osteoclast activity.

### Impaired Collagen Degradation After Rankl Induction in *mmp13b* Mutants

**FIGURE 3:**
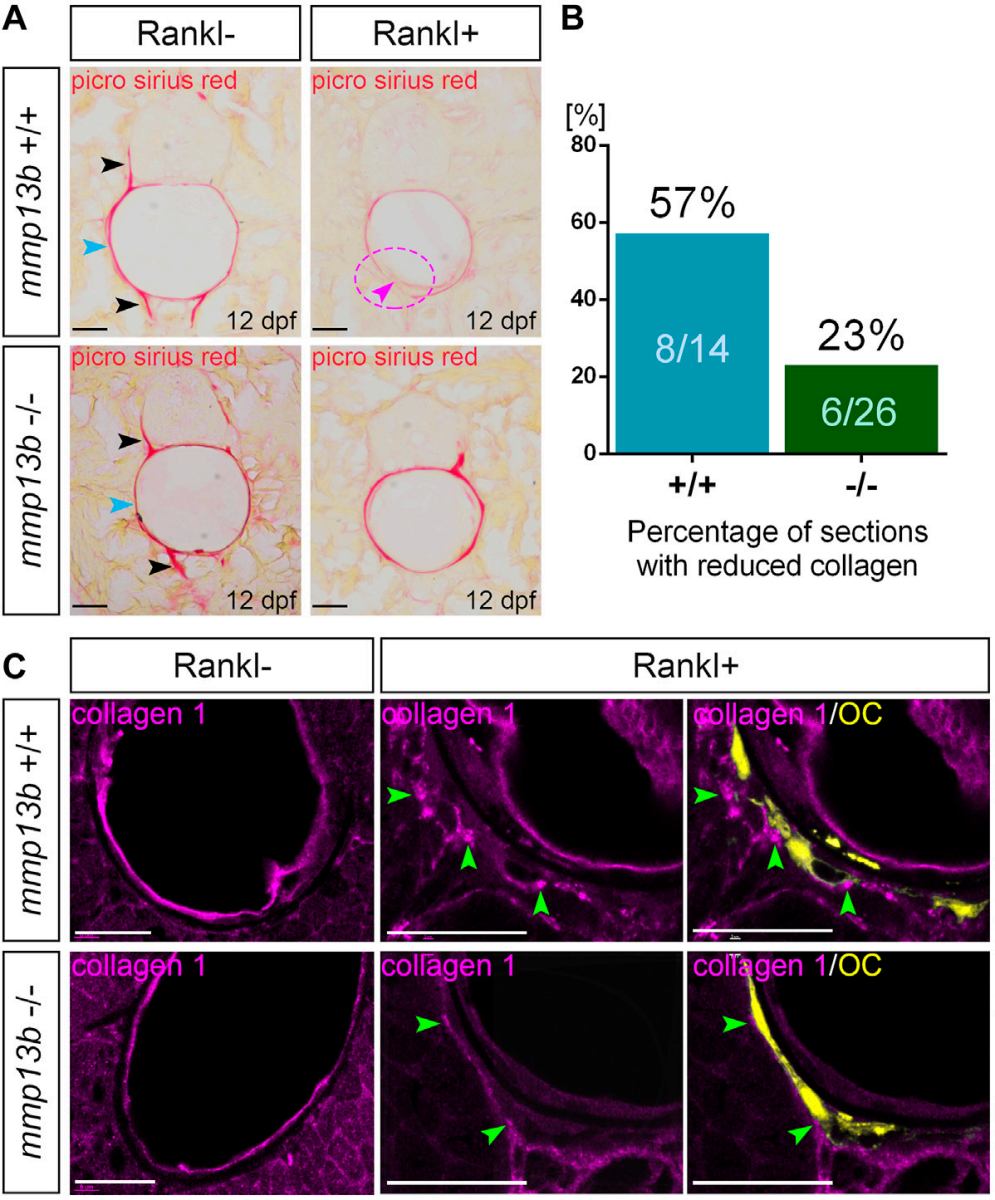
Impaired collagen degradation in *mmp13b* mutants after Rankl induction. **(A)** Picro sirius red stained cryosections from 12 dpf *mmp13b* ^+/+^ and *mmp13b* ^−/−^ larvae under Rankl- and Rankl+ conditions. Normal collagen distribution in neural arches and hemal arches (black arrows), and notochord surface (blue arrows) in *mmp13b* ^+/+^ and *mmp13b* ^−/−^ siblings under Rankl- condition. After Rankl induction (Rankl+), wildtype siblings but not homozygous mutants show reduced Picro sirius staining (magenta arrow and circle). **(B)** Quantification of the percentage of the sections with reduced Picrosirius in *mmp13b* ^+/+^ and *mmp13b* ^−/−^ after Rankl induction at 12 dpf. **(C)** Confocal images of transverse trunk sections from *mmp13b* ^+/+^ and *mmp13b* ^−/−^ larvae immunostained for collagen 1 (magenta) at 12 dpf. Without Rankl induction (Rankl-), collagen 1 protein is found along the outer surface of the notochord and notochordal sheath in *mmp13b* ^+/+^ and *mmp13b* ^−/−^ siblings. After Rankl induction (Rankl+), collagen 1 is degraded and transcytosed to the distal side of osteoclasts (yellow) in both *mmp13b* ^+/+^ and *mmp13b* ^−/−^ (green arrows). Transcytosed collagen 1 is less abundant in *mmp13b* ^−/−^ mutants. More than three larvae from each genotype were used for analysis. The section numbers used for each experiment are listed in Supplementary Table S4. Scale bar: 25 μm.

In medaka, blocking Mmp13b activity reduced Rankl-induced resorption of mineralized bone matrix. To investigate whether the degradation of non-mineralized collagen was also affected, a Picro-Sirius Red staining was conducted in *mmp13b* ^−/−^ mutants at 12 dpf to detect distribution of collagen fibres. During normal bone development (without Rankl induction, Rankl-), collagen staining was observed along the neural and hemal arches (Figure 3A; black arrowheads), and the outer surface of the notochord (blue arrowhead) in both wildtype siblings and homozygous mutants. In contrast, after Rankl induction, *mmp13b* ^+/+^ but not *mmp13b* ^−/−^ embryos showed strongly reduced collagen staining (Figure 3A; magenta arrowhead and circle), suggesting robust collagen degradation in wildtype siblings but not in *mmp13b* ^−/−^ mutants. A quantification revealed that more than half of the analyzed transverse sections (8/14 = 57%) showed reduced collagen staining for wildtype embryos, while a similar reduction was observed in only 23% of sections (6/26) from *mmp13b* ^−/−^ mutants (Figure 3B).

Collagen 1 is the major component of non-mineralized collagen fibres (Blair et al., 2016). Therefore, we performed Col1 immunostaining on transverse trunk sections of wildtype control siblings and *mmp13b* ^−/−^ embryos at 12 dpf (Figure 3C). Under non-osteoporotic Rankl- conditions, Col1 staining was observed along the outer surface of notochord and notochordal sheath in both wildtype siblings and *mmp13b* ^−/−^ mutants. In Rankl + embryos, on the other hand, Col1 was degraded and re-distributed to the distal side of osteoclasts in wildtype siblings (Figure 3C; green arrowheads), which is consistent with the process of transcytosis as described earlier (Salo et al., 1997; Ng et al., 2019). In *mmp13b* ^−/−^ embryos, however, redistributed Col1 fragments were significantly fewer in numbers when compared to wildtype siblings, indicating impaired degradation and transcytosis of Col1 in *mmp13b* mutants. Together, this suggests that Mmp13b is required for osteoclast resorption, including the degradation and transcytosis of non-mineralized collagens and resorption of mineralized bone matrix.

### Altered Morphology and Impaired Dynamics of Osteoclasts in *mmp13b* Mutants

**FIGURE 4:**
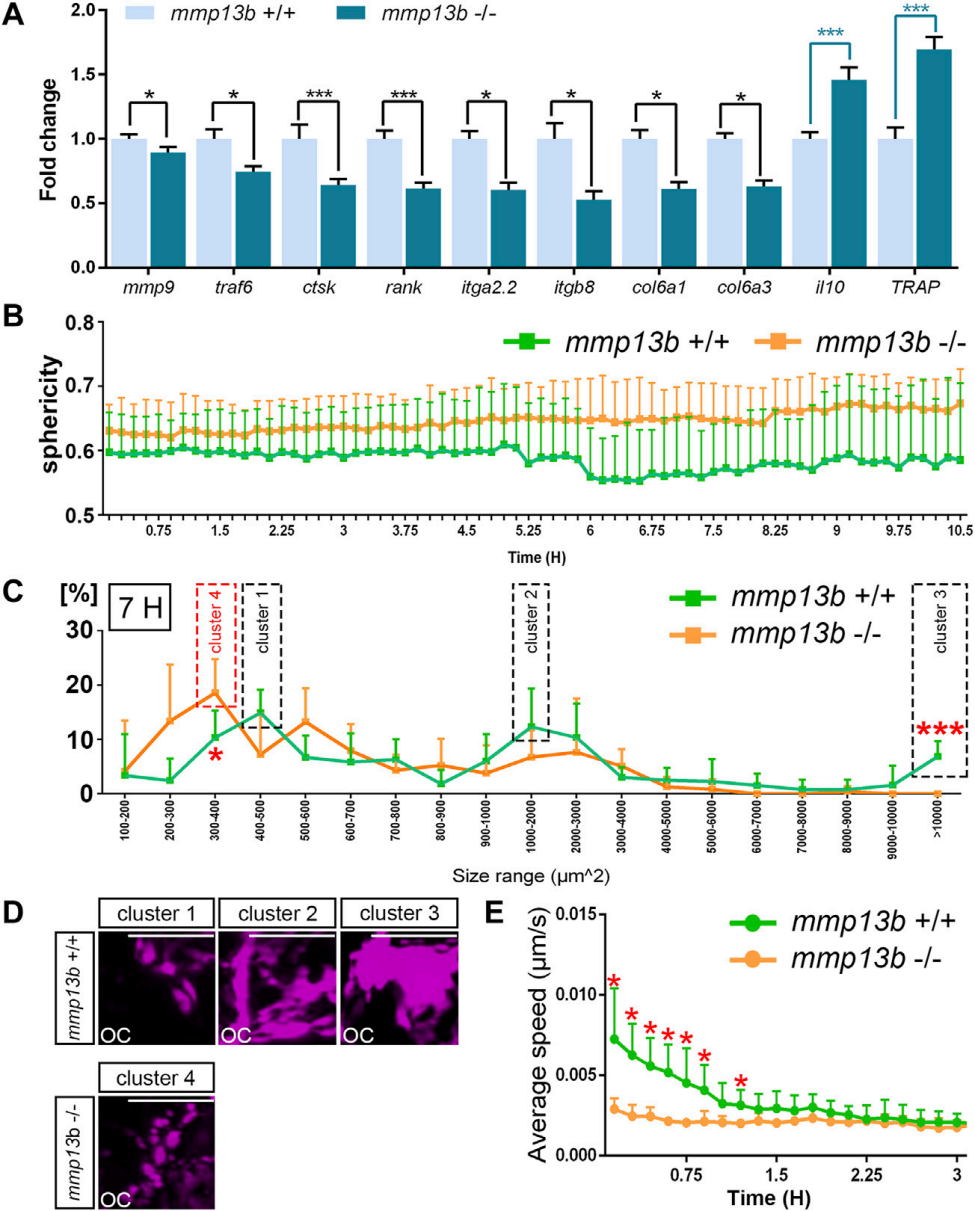
Osteoclast maturation and dynamics is impaired in *mmp13b* mutants. **(A)** Expression of differentiation genes in osteoclasts from *mmp13b* ^+/+^ and *mmp13b* ^-/-^ larvae at 3 dphs examined by qPCR. Significant down-regulation of *mmp9*, *traf6*, *ctsk*, *rank*, *itga2.2*, *itgb8*, *col6a1*, *col6a3,* and up-regulation of *il10* and *TRAP* in *mmp13b* ^-/-^ osteoclasts compared to *mmp13b* ^+/+^. **(B)** Quantification of osteoclast sphericity in *mmp13b* ^+/+^ and *mmp13b* ^-/-^ siblings. Compared to *mmp13b* ^+/+^, osteoclasts in *mmp13b* ^-/-^ show larger sphericity, indicating a rounder morphology in mutants. Sphericity Ψ is the ratio of the surface area of a sphere (with the same volume as the given particle) to the surface area of the particle. $\Psi =\frac{\pi^{1/3}{\left(6V_{p}\right)}^{2/3}}{A_{p}}$ Vp = volume of the particle, Ap = surface area of the particle. **(C)** Quantification of the osteoclast area in *mmp13b* ^+/+^ and *mmp13b* ^-/-^ siblings at 7 hours after the time-lapse imaging had started (at around 55 hphs). In wildtype siblings, the osteoclast areas displayed three peaks, defined as cluster 1, cluster 2, and cluster 3. In *mmp13b* mutants, one peak was found, which was named cluster 4. *mmp13b* ^+/+^: N=5, *mmp13b* ^-/-^: N=5. **(D)** Representative images of osteoclasts in four different clusters at 7 h. Scale bar: 50 μm. (E) Quantification of the osteoclast moving speed in wildtype siblings and homozygous mutants. In the first 1.5 hours of the time-lapse imaging analysis (about 48-49.5 hphs), osteoclasts in *mmp13b* ^-/-^ migrate significantly slower than that in *mmp13b* ^+/+^ siblings. The speed is the instantaneous speed of the object S(t), $S\left(t\right)=\frac{\sqrt{D_{x}{\left(t,t-1\right)}^{2}+D_{Y}{\left(t,t-1\right)}^{2}+D_{Z}{\left(t,t-1\right)}^{2}}}{T\left(t\right)-T\left(t-1\right)}$ . $mmp13b^{+/+}:N=6$ , $mmp13b^{-/-}:N=5$ . Error bars indicate mean ± SD, *P<0.05, **p<0.01, ***p<0.001. Unpaired Student’s two-tailed t test. All equations were taken from the Imaris Reference Manual.

Our findings suggested that osteoclasts form normally but show impaired resorption activities in *mmp13b* mutants. To understand the causes of reduced activity, we analyzed the maturation and migratory dynamics of osteoclasts in *mmp13b* mutants. First, *ctsk*-positive osteoclasts were isolated by FACS from *mmp13b* ^+/+^ and *mmp13b* ^−/−^ embryos at 12 dpf, and qPCR was conducted to examine transcription levels of several osteoclast differentiation genes, which we had earlier identified in medaka as upregulated under Rankl+ conditions (Phan et al., 2020a). These included *mmp9*, *rank*, *ctsk*, *traf6*, *TRAP*, and *il10*, as well as ECM-related genes, such as *col6a1*, *col6a3*, *itga2.2*, and *itgb8* (Figure 4A). Compared to wildtype siblings, *mmp9, rank, ctsk,* and *traf6* were significantly down-regulated in osteoclasts of *mmp13b* ^−/−^ mutants. Furthermore, *il10*, which is down-regulated in differentiated osteoclasts, had significantly increased transcription levels in *mmp13b* ^−/-^ osteoclasts. Together, this suggests that osteoclasts had retained an immature state and failed to fully differentiate in *mmp13b* mutants. Also, *col6a1*, *col6a3*, *itga2.2*, and *itgb8* were significantly down-regulated in osteoclasts of *mmp13b* ^−/−^ mutants, opening the possibility that bone cell-ECM interactions are impaired in mutant embryos. Surprisingly, the only osteoclast marker that we found up-regulated in *mmp13b* mutants, was *TRAP*. Consistently, also enzymatic TRAP activity could be detected in *ctsk*-positive cells by histochemical staining in transverse sections of *mmp13b* ^−/−^ embryos, while their distribution appeared affected (Supplementary Figure S3).

Next, to assess morphology and migratory dynamics of osteoclasts during bone resorption, live time-lapse imaging was conducted in wildtype siblings (Supplementary Movie S1) and *mmp13b* ^−/−^ mutants (Supplementary Movie S2) with a *rankl*:HS:*cfp*/*ctsk*:mCherry background starting from 48 h post heat-shock (hphs) at 11 dpf. Based on these movies, we determined sphericity, area, and migratory speed of osteoclasts. Compared to wildtype, osteoclasts in *mmp13b* ^−/−^ mutants generally exhibited higher sphericity (Figure 4B). At around 55 hphs (7 h after time-lapse imaging started), we observed that *ctsk*-positive cells generally exhibit a range of varying cell sizes and surface areas, and that this range was altered in mutants. For wildtype, most *ctsk*-positive osteoclasts could be categorized into three major clusters defined by distinct surface areas. Cells could be categorized into cluster 1 (with a surface area of 400–500 μm^2^), cluster 2 (1,000–2,000 μm^2^), and cluster 3 (>10,000 μm^2^) (Figure 4C). Osteoclasts in cluster 1 appeared as small and round cells, cells in cluster 2 had an elongated morphology, and cells in cluster 3 were characterized by large extended surface areas (Figure 4D). In stark contrast, most *ctsk*-positive cells in *mmp13b* mutants displayed a shift towards smaller area ranges, which we defined as cluster 4 (300–400 μm^2^) (Figure 4C), with rounder and smaller appearance (Figure 4D). Comparing osteoclasts in *mmp13b* ^−/−^ mutants with that in wildtype, we noticed a significant increase in the percentage of mutant cells in the 300–400 μm^2^ range and a significant decrease in the >10,000 μm^2^ range (Figure 4C). Finally, also the speed of dynamically moving osteoclasts was affected in mutants. In the first 1.5 h of time-lapse analysis (around 48–49.5 hphs), osteoclasts from wildtype siblings (N = 6) moved significantly faster than that from homozygous mutants (N = 5) (Figure 4E; and Supplementary Movies S1, S2). After 1.5 h, when the first highly active osteoclasts start to slow down to attach to bone matrix, there was no significant difference in the speed between wildtype and mutant osteoclasts. In summary, osteoclasts in *mmp13b* mutants exhibited signs of incomplete maturation upon Rankl induction when compared to wildtype siblings, suggesting that osteoblast-derived Mmp13b promotes osteoclast maturation and initiates osteoclast dynamics during bone remodeling.

### Elevated MMP Enzyme Activity in Osteoblasts Upon Rankl-Induction

**FIGURE 5:**
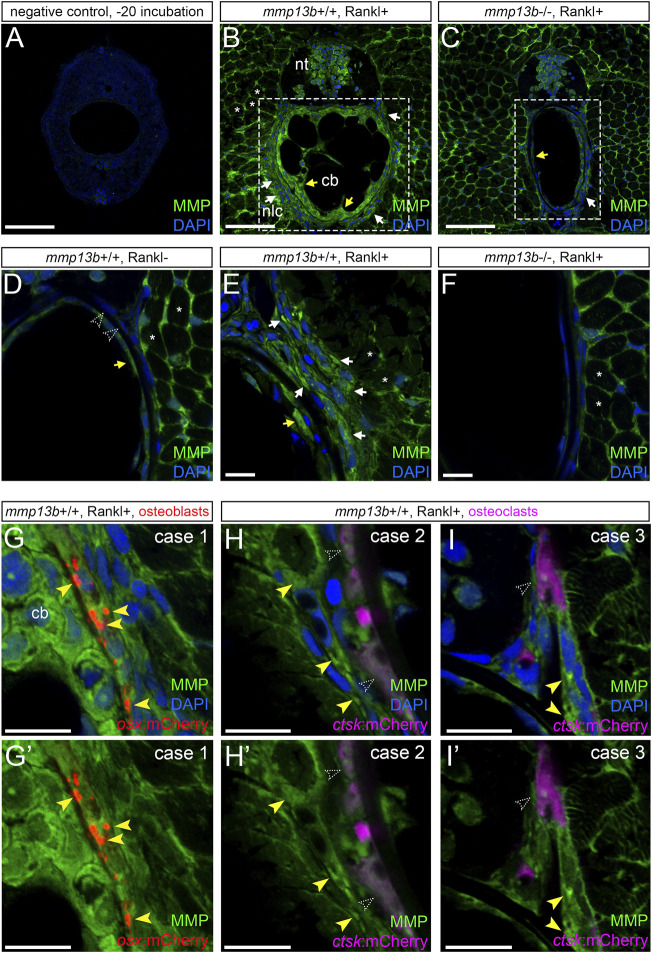
Analysis of MMP activity in *mmp13b* ^+/+^ and *mmp13b* ^−/−^ embryos using DQ collagen *in situ* zymography. *mmp13b* ^+/+^ and *mmp13b* ^−/−^ embryos with *rankl*:HS:*cfp*/*ctsk:*mCherry *or rankl*:HS:*cfp*/*osx:*mCherry transgenic background were heat-shocked at 9 dpf and transverse trunk sections were examined for enzymatic metalloprotease activity at 12 dpf. **(A)** Overview confocal images showing absence of MMP activity in control sections incubated at −20°C. **(B)** Strong MMP activity in *mmp13b* ^+/+^ along muscle fibres (asterisks), neural tube (nt), skeletal cells lining the outside of the notochord (white arrows) and chordoblasts (cb) inside the notochord (yellow arrows). **(C)** Reduced MMP activity in *mmp13b* ^−/−^ mutants in notochord lining cells (white arrow) and chordoblasts (yellow arrow). **(D)** High-magnification confocal images showing MMP activity under Rankl- conditions. No or little activity is detectable in chordoblasts (yellow arrow) or notochord lining cells (open arrowheads). **(E)** Increased MMP activity under Rankl + conditions in notochord lining cells (white arrows) and chordoblasts (yellow arrow). **(F)** *mmp13b* ^−/−^ mutants show similar MMP activity as *mmp13* ^*+/+*^ embryos under Rankl- conditions [as in **(D)**]. **(G)** MMP activity in notochord lining cells upon Rankl induction is mostly observed in *osx*:mCherry positive osteoblasts (yellow arrowheads; Case 1). **(H,H’)** Case 2: No or low MMP activity observed in *ctsk*:mCherry positive osteoclasts (open arrowheads) but strong activity in cells one to 2 cell diameters away (yellow arrowheads). **(I,I’)** Case 3: Osteoclast with low MMP activity (open arrowhead) in close contact to cells with high MMP (yellow arrowheads). **(G,H,I)** show nuclei stained with DAPI, while **(G’,H’,I’)** only show MMP activity and transgenic reporter expression. Scale bars: 50 μm **(A–F)**, 10 μm **(G–I’)**. More than three larvae from each genotype were used for analysis. The section numbers used for each experiment are listed in Supplementary Table S4.

Transcript levels for *mmp13b* were elevated upon Rankl induction as shown by RNAseq analysis and qPCR. To test whether upregulated RNA levels also translated into increased enzymatic metalloprotease (MMP) activity, we performed *in situ* zymography using a DQ-collagen substrate as described previously (Li et al., 2020). Control cryosections incubated at −20°C showed no or very little background enzymatic activity (Figure 5A). In contrast, strong enzymatic activity was observed in *mmp13b* ^*+/+*^ embryos after Rankl induction around muscle fibres, neural progenitors, chordoblasts inside the notochord and cells lining the notochord on the outside (Figure 5B). In *mmp13b* ^*−/−*^ mutants, on the other hand, enzymatic activity in chordoblasts and notochord-lining cells was markedly decreased, while levels in muscle fibres and neural progenitors remained high (Figure 5C). This suggests that enzymatic metalloprotease activity observed in cells of the vertebral column is mostly due to Mmp13b, which is consistent with high *mmp13b* transcript levels observed, e.g., by *in situ* hybridization (compare Figure 1E). Rankl-induction increased MMP activity in these cells in *mmp13b* ^*+/+*^ siblings but not *mmp13b* ^*−/−*^ mutants (Figures 5D–F). Next, we determined the identity of cells around the notochord that showed elevated enzymatic activity. For this, we performed analysis in transgenic lines expressing mCherry in *osx*-positive osteoblasts or *ctsk*-positive osteoclasts. This showed that notochord-lining cells with the highest enzymatic activity were osteoblasts (Figures 5G,G’), while osteoclasts had low levels of enzymatic MMP activity and were located one or 2 cell diameters away or directly adjacent to cells with high MMP activity (case 2 and 3, respectively; yellow arrowheads, Figures 5H,I).

Together, this strongly suggests that Rankl induction leads to elevated Mmp13b activity primarily in osteoblasts and that it is osteoblast-derived Mmp13b that promotes the maturation of adjacent osteoclasts and triggers their dynamic behavior required for bone remodeling.

### Fin Regeneration is Delayed in *mmp13b* Mutants

**FIGURE 6:**
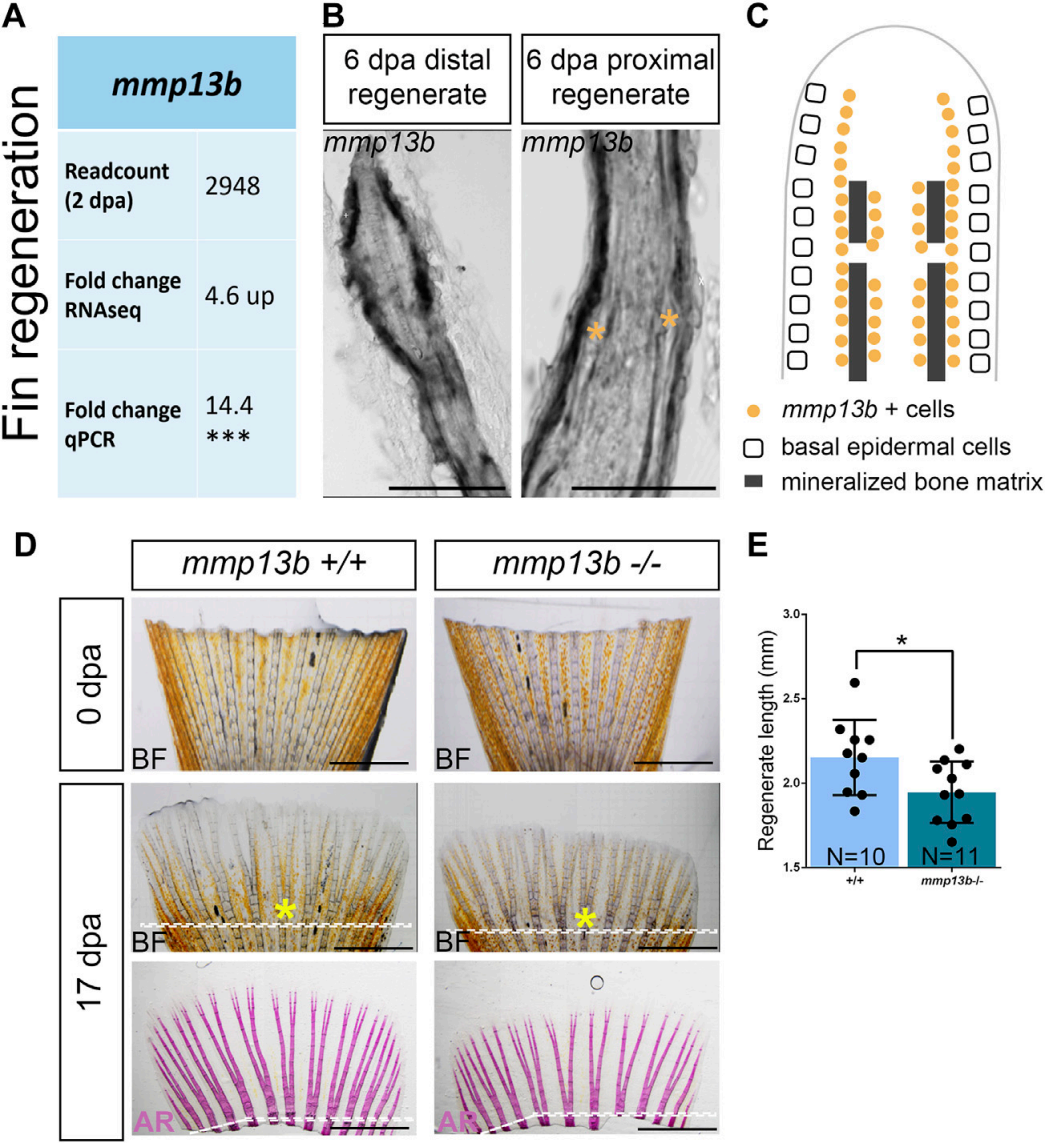
Delayed fin regeneration in *mmp13b* mutants at adult stage. **(A)** Table summarizing *mmp13b* readcount and fold change values obtained by RNAseq and qPCR analysis, respectively. *mmp13b* is up-regulated in osteoblast progenitors (*col10a1*) at 2 dpa compared to 0 dpa. **(B)** Confocal images of *mmp13b in situ* hybridization in longitudinal sections from 6 dpa wildtype fins. *mmp13b* expression is found in osteoblasts in the distal regenerated fin, and osteoblasts and joints (yellow asterisk) in the proximal regenerated fin. Scale bar: 50 μm. **(C)** Schematic diagram indicating *mmp13b* expression pattern at the distal and proximal region of a fin regenerate at 6 dpa in a longitudinal view. **(D)** Representative images of 0 and 17 dpa adult *mmp13b* ^+/+^ and *mmp13b* ^−/−^ fins. The amputation plane is denoted by a white dotted line. The sixth fin ray is marked by a yellow asterisk. *mmp13b* ^−/−^ mutants show delayed fin regeneration compared to wildtype siblings. Alizarin Red stained fins exhibit normal mineralization of bony structures in adult *mmp13b* ^+/+^ and *mmp13b* ^−/−^ fins. Scale bar: 1 mm. **(E)** Quantification of the fin regeneration length at 17 dpa in adult *mmp13b* ^+/+^ and *mmp13b* ^*−/−*^ siblings. The regenerated length is defined as the length from the tip to the amputation plane in the sixth fin ray. Error bars indicate mean ± SD, *p < 0.05, ***p < 0.001. unpaired Student’s two-tailed *t* test.

RNAseq analysis suggested that among other *mmp* genes, *mmp13b* was strongly upregulated in *col10a1* expressing osteoblast progenitors in the regenerating caudal fin at 2 dpa (Figure 6A; Supplementary Table S2). RNA *in situ* hybridization was used to confirm that *mmp13b* was expressed in osteoblast progenitors along the hemiray segments (Figures 6B,C). We noticed that *mmp13b* is also expressed in joint cells of the regenerating proximal fins (asterisks in Figure 6B). To assess whether *mmp13b* is required for fin regeneration, the fins of 2–3-month-old mutant fish were amputated and the length of regenerates were measured at 17 dpa. Compared with wildtype siblings, the regenerate lengths were significantly shorter in homozygous mutants, however the bony fin rays were mineralized normally (Figures 6D,E). Taken together, *mmp13b* is expressed in osteoblast progenitors of fin ray segments and joints, and its knock-out resulted in delayed fin regeneration in medaka.

### A *mmp13b* Deficiency Results in Altered Extracellular Matrix Structure and Reduced Osteoblast Progenitor Numbers in Fin Ray Joints

**FIGURE 7:**
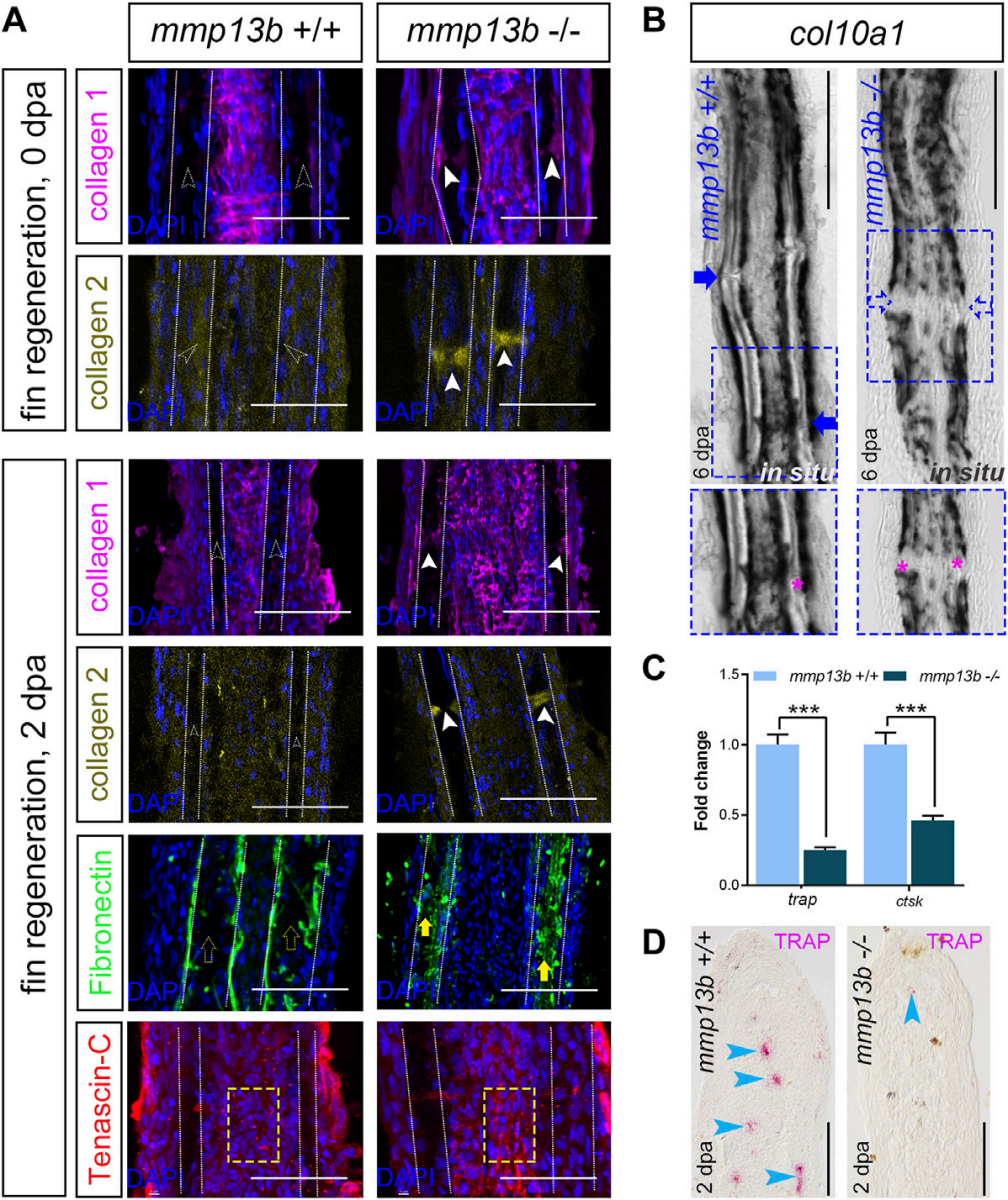
Altered ECM and reduced *col10a1* and TRAP expression in *mmp13b* mutant fins. **(A)** Confocal images of longitudinal sections from 0 to 2 dpa *mmp13b* ^+/+^ and *mmp13b* ^*−/−*^ fins immunostained with collagen 1 (magenta), collagen 2 (yellow), fibronectin (green) and tenascin-C (red). Compared to *mmp13b* ^+/+^ (hollow white arrows), excess collagen 1 and collagen 2 expression are found in joints in 0 and 2 dpa *mmp13b* ^−/*−*^ fins (white arrows). Enhanced fibronectin (yellow arrows) in the bone matrix region (between two white dotted lines) and more aggregated tenascin-C in the fibroblast-containing region (yellow box) in 2 dpa *mmp13b* ^−/*−*^ fins. Scale bar: 100 μm. **(B)** Overview and high-magnification confocal images of *col10a1 in situ* hybridization in longitudinal sections from 6 dpa *mmp13b* ^+/+^ and *mmp13b* ^*−/−*^ fins. *col10a1* is expressed in joints in *mmp13b* ^+/+^ but not *mmp13b* ^−/*−*^ fins (blue arrows). Joints are marked by magenta asterisk in zoom in images. **(C)** *ctsk* and *trap* qPCR in 2 dpa *mmp13b* ^+/+^ and *mmp13b* ^−/*−*^ fin samples. Error bars indicate mean ± SD, Student’s two-tailed *t* test, ***p < 0.001. **(D)** TRAP staining in longitudinal sections from 2 dpa *mmp13b* ^+/+^ and *mmp13b* ^−/*−*^ fins. TRAP positive cells (magenta arrows) are found in *mmp13b* ^+/+^ blastema, but not in *mmp13b* ^−/*−*^. More than three fins from each genotype were used for analysis. Section numbers used for experiment are listed in Supplementary Table S4.

We next assessed whether a deficiency in *mmp13b* alters the ECM structure in fins and thereby affects the efficiency of regeneration. We analyzed matrix proteins that are known substrates of Mmp13b, such as collagen 1 (Hayami et al., 2008), collagen 2 (Howes et al., 2014), fibronectin (Zhang et al., 2012), and tenascin-C (Yamamoto et al., 2016), in fin rays of *mmp13b* ^+/+^ and *mmp13b* ^−/−^ fish at 0 and 2 dpa. In control fins at 0 dpa, no presence of collagen 1 and collagen 2 was detected in joint regions (Figure 7A; empty arrowheads). In contrast, collagen 1 and collagen 2 were strongly expressed in joints of *mmp13b* ^−/−^ fins (Figure 7A; white arrowheads). This suggests that the collagen composition is altered in the joint regions of *mmp13b* mutants. Also, at 2 dpa, excess collagen 1 and collagen 2 was detectable in homozygous but not wildtype joints. In addition, also fibronectin showed enhanced expression in the bone matrix region in mutant fins (Figure 7A; yellow arrows) at 2 dpa when compared to wildtype (yellow empty arrows). For tenascin-C, in the fibroblast-containing region in the center of the fin ray (yellow box), its distribution appeared to be more aggregated in *mmp13b* ^−/−^ fins compared to *mmp13b* ^+/+^ fins. Taken together, these findings suggest that a *mmp13b* deficiency results in an altered ECM composition in fin rays. This includes an excess of matrix proteins especially in the joints, which harbor progenitor cells needed for regeneration (Ando et al., 2017; Dasyani et al., 2019). To examine whether an altered ECM structure in the joints of *mmp13b* ^*−/−*^ deficient fins affected the number of osteoblast progenitors, we analyzed expression of the progenitor marker *col10a1* by *in situ* hybridization in fins at 6 dpa. This showed that, as reported earlier (Dasyani et al., 2019), *col10a1* is expressed in joints of wildtype fins (Figure 7B, blue arrow). In contrast, in *mmp13b* ^−/−^ fins, *col10a1* expression in joints was strongly reduced (Figure 7B; blue box, blue arrows). This suggests that the number of *col10a1-*positive osteoblast progenitors is reduced in the joints of *mmp13b* deficient mutants.

Fin regeneration depends on the recruitment of *mpeg1*-positive macrophages to the amputation site (Petrie et al., 2014). These macrophages also express tartrate resistant acidic phosphatase (TRAP) and cathepsin K (*ctsk*) (Blum and Begemann, 2015). qPCR analysis showed that transcript levels of both *trap* and *ctsk* were significantly downregulated in *mmp13b* ^−/−^ fins at 2 dpa (Figure 7C). Consistent with this, also histochemical TRAP staining revealed a reduction or absence of TRAP-positive cells in the regeneration blastema of *mmp13b* ^−/−^ fins, when compared to wildtype (Figure 7D; blue arrowheads). We also analyzed distribution of *mpeg1*:mCherry/TRAP double-positive cells in regenerating fins at 2 dpa (Supplementary Figure S5). This revealed the presence of both *mpeg1*/TRAP double-positive cells as well as TRAP-positive/*mpeg1*-negative cells. We conclude that the latter are osteoclasts that have differentiated from macrophages and that therefore at least a subset of TRAP-positive cells are osteoclasts. Together, this suggests that a deficiency in Mmp13b results in a modified ECM composition in regenerating fins with a concomitant reduction in osteoblast progenitor and macrophage/osteoclast numbers, consequently leading to a delay in fin regeneration.

### *mmp9* ^−/−^;*mmp13b* ^−/−^ Double Mutants Exhibit an Osteopetrosis-Like Phenotype in Medaka

**FIGURE 8:**
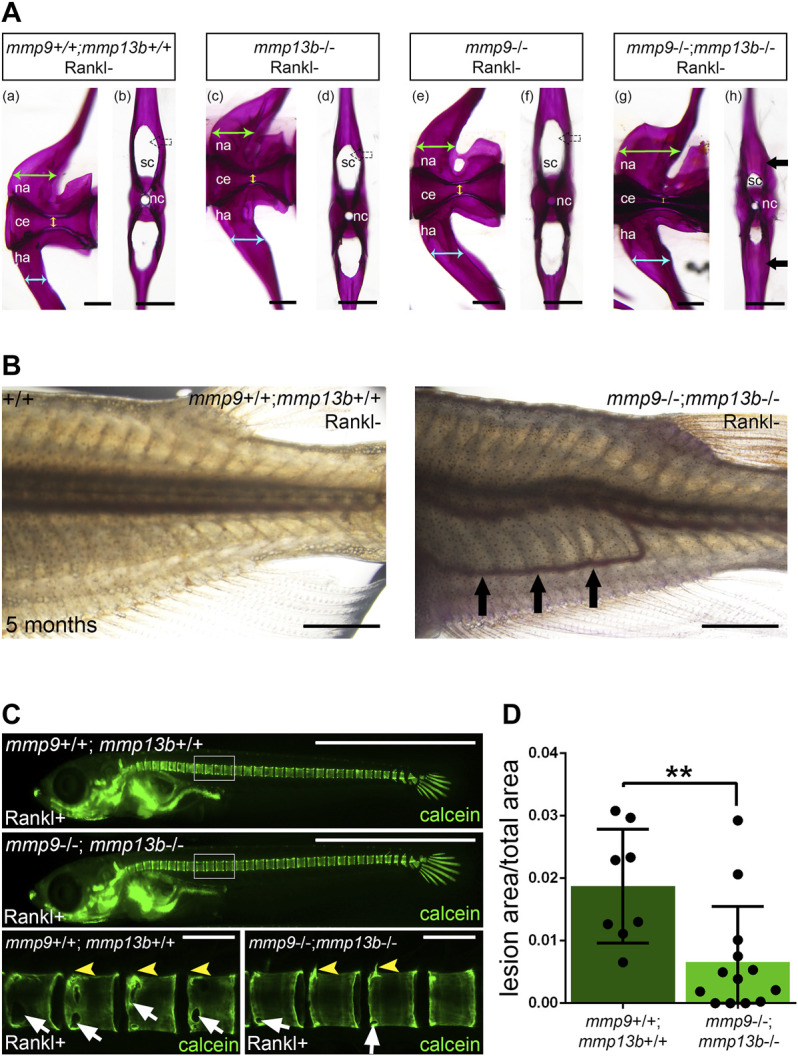
Excess bone in vertebral arches of *mmp9/mmp13b* double mutants. **(A)** High-magnification lateral and transverse views of Alizarin Red stained vertebrae of 5 month-old wildtype siblings (a, b), *mmp13b* ^−/−^ mutants (c, d), *mmp9* ^−/−^ mutants (e, f), and *mmp9* ^−/−^;*mmp13b* ^−/−^ double mutants (g, h). Green and cyan arrows indicate the width of neural and hemal arches, respectively. The diameter of the centra is denoted by yellow arrow. Black arrow indicates excessive mineralized bone matrix in neural arch of *mmp9* ^−/−^;*mmp13b* ^−/−^ double mutant (h). na: neural arch; ce: centra; ha: hemal arch; sc: spinal cord; nc: notochord. Scale bar: 200 μm. **(B)** Bright field images showing the trunk of wildtype siblings and *mmp9* ^−/−^;*mmp13b* ^−/−^ mutant at 5 months. Ectopic blood vessel formation is denoted by black arrows. Scale bar: 2 mm. **(C)** Overview and high-magnification confocal images of calcein stained mineralized matrix in Rankl transgenic controls and *mmp9*−/−; *mmp13b*−/− siblings at 3 days after Rankl induction (3 dphs; 12 dpf). Control siblings but not homozygous mutants show enhanced resorption of mineralized matrix at neural arches (yellow arrowheads) and vertebral bodies (white arrows). **(D)** Quantification of bone lesions in vertebral column and neural arch [region marked as white boxes in **(C)**] in controls and *mmp9*−/−; *mmp13b*−/− siblings at 3 dphs. +/+: N = 8, *mmp9*−/−; *mmp13b*−/−: N = 13. Error bars indicate mean ± SD, **p < 0.01, unpaired Student’s two-tailed *t* test. Scale bar: 1 mm (overview images), 100 μm (zoom in images).

Medaka *mmp13b* ^*−/−*^ mutants develop normally into adulthood with no obvious deficiencies in bone remodeling. This suggests that other Mmps compensate for the loss of Mmp13b, similar to the situation in mice. There, *Mmp9* ^−/−^;*Mmp13* ^−/−^ double mutants exhibited more severe defects in growth plate formation and bone development compared to the respective single mutants (Stickens et al., 2004). Interestingly, our RNAseq data showed strong expression and up-regulation of *mmp9* in osteoblast progenitors and osteoblasts after Rankl induction (Supplementary Table S1). As this opened the possibility of a synergism with *mmp13b*, we generated *mmp9* ^−/−^;*mmp13b* ^−/−^ double mutants in medaka to identify potential compensatory effects on endogenous bone remodeling. At embryonic stages, *mmp9* ^−/−^;*mmp13b* ^−/−^ double mutants developed normally. However, severe bone defects were observed in adult mutants at 5 months. Alizarin red staining revealed normally mineralized bone in wildtype controls and *mmp9* ^−/−^ as well as *mmp13b* ^−/−^ single mutants. In contrast, *mmp9* ^−/−^;*mmp13b* ^−/−^ double mutants showed an increase of mineralized bone mass in the arches of vertebral bodies (Figure 8A). Transverse views of single vertebral bodies revealed excess bone matrix in the neural arches of *mmp9* ^−/−^;*mmp13b* ^−/−^ mutants, reducing the size of the canal that contains the spinal cord (Figure 8A; black arrow). Also, hemal arches had increased bone mass resulting in an obstruction of the hemal canal and dislocation of blood vessels (Figure 8B; black arrows). This phenotype was likely caused by reduced bone resorbing activity of the normally formed osteoclasts, as evident upon Rankl induction in *mmp9* ^−/−^;*mmp13b* ^−/−^ double mutants (Figures 8C,D; Supplementary Figure S4). Together, this suggests that *mmp9* and *mmp13b* work redundantly and are required for activity of endogenous osteoclasts as well as under osteoporotic conditions. Interestingly, *mmp9* ^−/−^;*mmp13b* ^−/−^ double mutants also showed delays in fin regeneration (Supplementary Figure S5).

## Discussion

Osteoblast and osteoclast activities during bone remodeling require coordinated control to achieve homeostasis and prevent the loss of bone mineral density. Various signaling pathways have been identified that control bone cell interactions (Nakashima et al., 2002; Luo et al., 2016; Phan et al., 2020b). Besides communication through cell signaling, contact-dependent cell activation also occurs through alteration of structural components of the ECM (Delaissé et al., 2003; reviewed by Paiva and Granjeiro, 2014; reviewed by Pedersen et al., 2015). In our study, we characterized the matrix metalloprotease Mmp13b in medaka and showed that it is required for osteoclast maturation and determines the morphology and dynamics of osteoclasts under osteoporotic conditions. In addition, Mmp13b also controls the composition of ECM during fin regeneration and contributes to the recruitment of progenitor cells to regenerating tissue.

### Osteoblast-Derived Mmp13b Regulates Osteoclast Driven Resorption in a Non-cell Autonomous Manner

Transcriptome profiling of bone cells under osteoporotic conditions revealed that two *mmp13* orthologs, *mmp13* (ENSORLG00000020553) and *mmp13b* (ENSORLG00000014453) are expressed in medaka. The zebrafish genome also has two *mmp13* genes, *mmp13a* (ENSDARG00000012395) and *mmp13b* (ENSDARG00000100794). Earlier studies have focused on *mmp13a,* as *mmp13b* is expressed at low levels and transcripts cannot be detected by qPCR (Zhang et al., 2017) or *in situ* hybridization (Li et al., 2020)*.* Zhang et al. (2017) reported that *mmp13a* was up-regulated in *atp6v1h*-deficient zebrafish that have an osteoporosis-like phenotype suggesting a potential role in bone resorption.

In medaka, on the other hand, *mmp13a* (annotated as *mmp13* in ENSEMBL, see above) showed low expression levels in osteoblasts and was not differentially regulated upon Rankl induction (Supplementary Table S1). Therefore, we focused our study on *mmp13b* and investigated its function in bone formation and remodeling. RNA *in situ* hybridization confirmed that *mmp13b* is expressed in osteoblast progenitors and osteoblasts. In addition, *in situ* zymography confirmed strongly elevated MMP enzymatic activity in osteoblasts upon Rankl induction. The MMP activity was strongly reduced in *mmp13b* mutants. Together, this suggests that osteoblast progenitors and osteoblasts produce enzymatically active Mmp13b.

Its expression in osteoblasts could reflect a possible role for Mmp13b in bone formation. Such a role was previously shown in mice where Mmp13 is expressed in hypertrophic chondrocytes and osteoblasts and contributes to endochondral bone ossification and fracture healing (Yamagiwa et al., 1999; Stickens et al., 2004). In *Mmp13* ^−/−^ mice, hypertrophic chondrocytes exhibited a delayed exit from the growth plate and accumulated in a terminally differentiated state, leading to an expanded hypertrophic chondrocyte zone (Inada et al., 2004; Stickens et al., 2004). In addition, osteoblast-derived Mmp13 is responsible for proper cartilage degradation and its inactivation results in increased trabecular bone in mice (Stickens et al., 2004). Beyond normal bone development, *Mmp13* also promoted fracture healing through degrading cartilage ECM components, such as aggrecan (Kosaki et al., 2007). In our study, however, *mmp13b* mutant embryos developed normally suggesting that this matrix metalloprotease is dispensable for normal bone formation. Other Mmps could compensate for the loss of *mmp13b* in bone formation. Interestingly, however, *mmp9*, which among other *mmp* genes, was the most strongly expressed and upregulated in osteoblasts under osteoporotic conditions (Supplementary Table S1), does not appear to compensate for the loss of *mmp13b* in bone formation. Medaka *mmp9*−/−;*mmp13b*−/− double mutant embryos developed normally suggesting that these proteases are not required for regular bone formation. Instead, *mmp9*−/−;*mmp13b*−/− double mutants exhibited severely increased bone mass in the vertebral bodies at 5 months, with an obstruction of neural and hemal canals in the vertebral arches leading to a dislocation of blood vessels. This high bone mass phenotype is intriguingly similar to the osteopetrosis-like symptoms that we had earlier reported for an osteoclast-deficient medaka model (To et al., 2015). This thus strongly suggests that *mmp9* and *mmp13b* act synergistically to control the activity of endogenous osteoclasts and control bone remodeling at adult stages.

Medaka *mmp13b* single mutants instead showed defects under osteoporosis-like conditions. After Rankl induction, ectopic osteoclast formed normally but bone resorption was reduced in *mmp13b* ^−/−^ mutants, and this reduction could be recapitulated by chemical Mmp13 inhibition. This strongly suggests that Mmp13b plays a critical role in osteoclast activation under osteoporotic conditions. The role of *Mmp13* in regulating osteoclast differentiation and resorption has been studied in various tumor models. For example, MMP13 was found to be induced in breast tumors (Freije et al., 1994), where its up-regulation at the interface between tumor tissue and bone matrix contributed to bone metastasis through promoting osteoclast differentiation (Nannuru et al., 2010; Pivetta et al., 2011). Another study showed that myeloma-derived MMP13 regulates the fusion of osteoclasts and bone resorption (Fu et al., 2016). This shows that MMP13 produced by tumor cells activates osteoclasts and promotes bone metastasis.

In medaka, we demonstrated that the maturation of osteoclasts depends on Mmp13b. We analyzed ten known osteoclast-relevant genes that were earlier shown to be upregulated when macrophages differentiate into mature osteoclasts (Phan et al., 2020a). In *mmp13b* mutants, these genes were down-regulated after Rankl induction while *il10*, a known osteoclast inhibitor (Evans and Fox, 2007; Zhang et al., 2014) was up-regulated. Together, this strongly suggests that Mmp13b is required for the maturation of osteoclasts under osteoporosis-like conditions. However, the observation that *trap*, a well-established osteoclast marker (Andersson and Marks, 1989), was up-regulated indicates that osteoclast differentiation was not entirely blocked in *mmp13b* mutants, although bone resorption was strongly reduced.

Also, the morphology of osteoclasts was affected in *mmp13b* mutants. Osteoclasts showed a larger sphericity in *mmp13b* ^−/−^ mutants when compared to wildtype, which could reflect the more immature state of these cells. In addition, also the size or surface area of osteoclasts was affected in *mmp13b* mutants. In wildtype embryos, *ctsk*-positive osteoclasts were found to exhibit three distinct size ranges, which we defined as immature (cluster 1), mono-nucleated (cluster 2) or multi-nucleated osteoclasts (cluster 3). Importantly, in *mmp13b* mutants, a fourth size range (cluster 4) was detected, which was similar to cluster 1 and consistent with the idea that these cells were immature. Finally, also the motility of *ctsk* cells was affected in *mmp13b* mutants. In wildtype siblings, osteoclasts exhibit a dynamic motility pattern, with a relatively fast speed (approx. 8 nm/s) in the first 1.5 h of the time-lapse analysis (around 48–49.5 hphs), which is consistent to earlier reports (Phan et al., 2020a), followed by a slowing down to a consistent speed of approx. 2 nm/s. This change in speed might reflect the transition of an immature osteoclast approaching the vertebral column to a differentiated osteoclast making contact to mineralized matrix (Phan et al., 2020a). In *mmp13b* mutants, on the other hand, no elevated speed was observed suggesting possible deficiencies in motility of the immature osteoclasts. In conclusion, our findings on expression profiles, sphericity, size and motility strongly suggest that osteoclasts fail to fully mature in *mmp13b* mutants.

Similar osteoclast defects were observed in medaka embryos treated with the MMP13 inhibitor CL-82198 hydrochloride, which had earlier been used for the treatment of osteoarthritis (Wang et al., 2013). These inhibitor experiments thus confirmed our mutant analysis and demonstrate that the medaka model can be used for future screening of compounds that interfere with Mmp13 activity to potentially prevent osteoporosis or tumor-caused osteolysis.

In mammals, MMP13 has similar activities in osteocytes and osteoblasts. In both cell types, MMP13 activity is required to remove and remodel the surrounding ECM. On the other hand, our study suggests that in the anosteocytic bone of medaka, osteoblast-derived Mmp13b in addition functions in a non-cell autonomous manner to activate osteoclasts. Whether in osteocyte-inhabited bone, osteocyte-derived MMP13 plays similar roles in osteoclast activation remains to be investigated.

### Mmp13b Controls Extracellular Matrix Remodeling During Medaka Fin Ray Regeneration

Comparing the transcriptome profiles of osteoblasts during bone remodeling under osteoporosis-like conditions and fin regeneration, we identified a significant up-regulation of *mmp13b* in *col10a1* positive osteoblast progenitors. These *col10a1* cells contribute substantially to the regeneration of fin ray segments and joints in medaka (Dasyani et al., 2019). RNA *in situ* hybridization confirmed *mmp13b* expression in osteoblasts of segments and fin ray joints. Earlier studies had reported the important role of joint cells in fin regeneration. In zebrafish, *mmp9*-expressing joint cells represent a progenitor pool for fin regeneration (Ando et al., 2017). Like the situation in *mmp9* mutant zebrafish, in our study, we also observed delayed fin regeneration in medaka *mmp13b* ^−/−^ single and *mmp9* ^−/−^;*mmp13b* ^−/−^ double mutants. While being expressed in osteoblast progenitors, we propose a direct role for Mmp13b protein in generating an ECM environment that promotes regeneration.

*Mmp13* has earlier been implicated in regeneration processes, including skeletal (Behonick et al., 2007), muscle (Lei et al., 2013), heart (Xu et al., 2018), and fin regeneration (Li et al., 2020). In zebrafish, a knock-down of *mmp13a* resulted in delayed fin growth and impaired osteoblast differentiation (Li et al., 2020). Hence, our findings in stable *mmp13b* knock-out medaka mutants are consistent with morpholino-based findings in zebrafish.

During fin regeneration, the ECM in the blastema is undergoing extensive remodeling and different matrix proteins are dynamically produced and degraded to generate a distinct ECM environment (Mercer et al., 2012; Govindan and Iovine, 2015). For example, hyaluronic acid (HA), fibronectin (FN) and tenascin C (TNC) are up-regulated to form a transitional matrix for cell proliferation and differentiation during early regeneration stages (Calve et al., 2010). On the other hand, collagen expression is rapidly reduced (Govindan and Iovine, 2015). In our study, we analyzed expression levels and distribution of collagen 1, collagen 2, fibronectin, and tenascin-C in the regenerating fin rays. We found that in regenerating wildtype fins at two stages (0 and 2 dpa), almost no collagen 1 and collagen 2 protein was detectable in the joint regions. This contrasted with *mmp13b* mutants, where excess collagen 1 and collagen 2 was observed in joints, suggesting impaired collagen degradation when Mmp13b activity is reduced. Two matrix proteins important for the transitional ECM, fibronectin and tenascin-C, also exhibited altered distribution in wildtype siblings and *mmp13b* mutants. At 2 dpa, fibronectin was upregulated in both wildtype and *mmp13b* mutants when compared to 0 dpa (data not shown). However, compared to wildtype where fibronectin was found along bone lining osteoblasts, enhanced fibronectin levels were observed in the bone matrix region in *mmp13b* mutants. Finally, tenascin-C appeared more aggregated in the fibroblast-containing region of *mmp13b* mutants when compared to wildtype. In addition to these ECM changes, we also observed reduced numbers of *col10a1*-positive osteoblast progenitors in newly formed joint regions of mutants. Insufficient numbers of progenitors in the joint regions are expected to delay fin regeneration, as joints have been identified as the reservoir of cells needed for fin regeneration (Ando et al., 2017). Together, we hypothesize that Mmp13b is needed to remodel the ECM and allow recruitment of osteoblast progenitors to the joints where they are needed for regeneration.

The initial phases of fin regeneration in zebrafish depend on the recruitment of macrophages (Nguyen-Chi et al., 2017), while at later stages TRAP-positive osteoclasts are detected in the regenerating fins where they contribute to remodeling (Blum and Begemann, 2015; Ando et al., 2017). In our study, we also observed TRAP-positive cells in the regenerating fins at an early stage. While we are unable to distinguish whether these TRAP-positive cells represent macrophages or osteoclasts, no such cells were observed in *mmp13b* mutants. We thus conclude that remodeling the ECM through Mmp13b is not only required for recruitment of osteoblasts but also of other cell types needed for regeneration.

In conclusion, our study using transcriptome profiling in a medaka model has identified Mmp13b as an important regulator needed for ECM remodeling during tissue regeneration and for maturation of osteoclasts during osteoporotic conditions. The fact that *mmp13b* was found strongly expressed in osteoblast progenitors in both situations implies interesting scenarios involving cell autonomous and non-cell autonomous roles, respectively. While in regeneration, *mmp13b*-positive osteoblasts seem to remodel the ECM to attract more osteoblasts into the joint regions, under osteoporotic conditions, osteoblast-derived Mmp13b controls maturation of osteoclasts. Whether the latter is also through ECM remodeling or alternatively through proteolytic activation of other factors needed for osteoclast maturation, remains to be analyzed.

## Data Availability

The data presented in the study are deposited in the NCBI Sequence Read Archive repository, accession number PRJNA767186.

## Supplementary Material

Supplementary Figure S1 Transcriptome profiles of medaka osteoblast progenitors in 0 and 2 dpa fins. **(A)** Venn diagram showing the total number of genes expressed in *col10a1*-positive osteoblast progenitors in 0 and 2 dpa fins, with the overlapping region showing commonly expressed genes. **(B)** Volcano plot showing up- and downregulated genes in osteoblast progenitors at 2 dpa compared with 0 dpa with adjusted p values <0.05 (*p* adj value <0.05). Insignificantly regulated genes are shown as blue dots. **(C)** KEGG pathway enrichment analysis of differentially expressed genes (DEGs) showing significantly enriched categories including extracellular matrix-receptor interaction (red box). KEGG, Kyoto Encyclopedia of Genes and Genomes; *q* value, adjusted *p* value.

Supplementary Figure S2 Generation of *mmp13b* mutants. Generation of *mmp13b* CRISPR/Cas9 mutants using three guide RNAs (gRNA1-3) targeting exon 2 (E2), exon 3 (E3) and exon 6 (E6). Magenta arrows indicate position of forward (FP) and reverse (RP) primers for PCR genotyping [WT (+/+): 1250 bp; homozygous mutants (−/−): 816 bp; heterozygous carriers (+/−): 1250 bp and 816 bp]. Mutants have a 434 bp deletion (yellow) between gRNA2 and gRNA3 target sites, leading to a predicted prematurely truncated aa sequence (red).

Supplementary Figure S3 Presence of TRAP-positive cells upon Rankl induction in *mmp13b* ^−/−^ mutants. TRAP staining on transverse sections of trunk of 12 dpf *mmp13b* ^+/+^ and *mmp13b* ^−/−^ mutants with *ctsk*:mCherry transgenic background after Rankl induction. Normal formation but uneven distribution (hollow arrow) of TRAP-positive cells in *mmp13b* ^−/−^ mutants compared to *mmp13b* ^+/+^. More than three larvae of each genotype were used for cryosectioning. Section numbers: *mmp13b* ^+/+^: N=23; *mmp13b* ^−/−^: N=30. Scale bar: 100 μm.

Supplementary Figure S4
*mmp9*/*mmp13b* double mutants show normal osteoclast formation after Rankl induction. Overview and high-magnification confocal images of controls (+/+) and *mmp9* ^−/−^; *mmp13b* ^−/−^ double mutants in *rankl*:HS:*cfp*/*ctsk*:mCherry transgenic background at 2 dphs (11 dpf). Similar osteoclast formation in +/+ and *mmp9* ^−/−^; *mmp13b* ^−/−^ at 2 dphs.

Supplementary Figure S5 Distribution of *mpeg1*:mCherry and TRAP-positive cells in regenerating fins at 2 dpa. Caudal fins of 3 month-old adult medaka with *mpeg1*:mCherry transgenic background were amputated and 2 dpa fins were collected for TRAP staining. Overview fluorescence images **(A,E)** showing presence of *mpeg1*-positive macrophages in blastema before TRAP staining. Zoom in images [of areas indicated by white boxes in **(A,E)**] showing TRAP-positive cells **(C,G)** and mCherry-positive cells before **(B,F)** and after **(D,H)** TRAP staining. White and cyan arrowheads show *mpeg1*-positive and *mpeg1*/TRAP double positive cells, respectively. Blue arrows indicate TRAP-positive but *mpeg1*-negative cells. Scale bar: 50 μm **(A,E)**, 25 μm **(B–D, F–H)**.

Supplementary Figure S6
*mmp9* ^−/−^; *mmp13b* ^−/−^ double mutants exhibit mild but distinct delay in fin regeneration at 17 dpa. **(A)** Representative images of fins at 17 dpa in adult wildtype controls (+/+) and *mmp9* ^-/-^; *mmp13b* ^−/−^ double mutants. The amputation plane is denoted by a red dotted line. *mmp9* ^−/−^; *mmp13b* ^−/−^ mutants show delayed fin regeneration compared to wildtype siblings. Scale bar: 1 mm. **(B)** Quantification of the fin regeneration length at 17 dpa in adult +/+ and *mmp9* ^−/−^; *mmp13b* ^−/−^ siblings. The regenerated length is defined as the length from the tip to the amputation plane in the 6th fin ray. +/+: N=11, *mmp9* ^−/−^; *mmp13b* ^−/−^: N=8. Error bars indicate mean ± SD, ***p* < 0.01, unpaired Student’s two-tailed *t* test.

Supplementary Movie S1 Time-lapse analysis of osteoclast dynamics in wildtype siblings. Larvae with *rankl*:HS:*cfp*/*ctsk*:mCherry transgenic background were heat-shocked at 9 dpf (2 h, 39°C) and time-lapse imaging started at 48 hphs. Osteoclasts show dynamic morphology. Scale bar: 50 μm. Time: hh:mm:ss. Frame rate: 10 fps.

Supplementary Movie S2 Time lapse analysis of osteoclast dynamics in *mmp13b* ^−/−^ mutants. Mutants with *rankl*:HS:*cfp*/*ctsk*:mCherry transgenic background were heat-shocked at 9 dpf (2 h, 39°C) and time-lapse imaging started at 48 hphs. Osteoclasts in mutants exhibit altered morphology and dynamics. Scale bar: 50 μm. Time: hh:mm:ss. Frame rate: 10 fps.
