# Supplementary figures and images for: Transcriptome Profiling of Osteoblasts in a Medaka (Oryzias latipes) Osteoporosis Model Identifies Mmp13b as Crucial for Osteoclast Activation
Source: Front Cell Dev Biol. 2022 Feb 21;10:775512. doi: 10.3389/fcell.2022.775512 (PMC8911226; doi:10.3389/fcell.2022.775512)

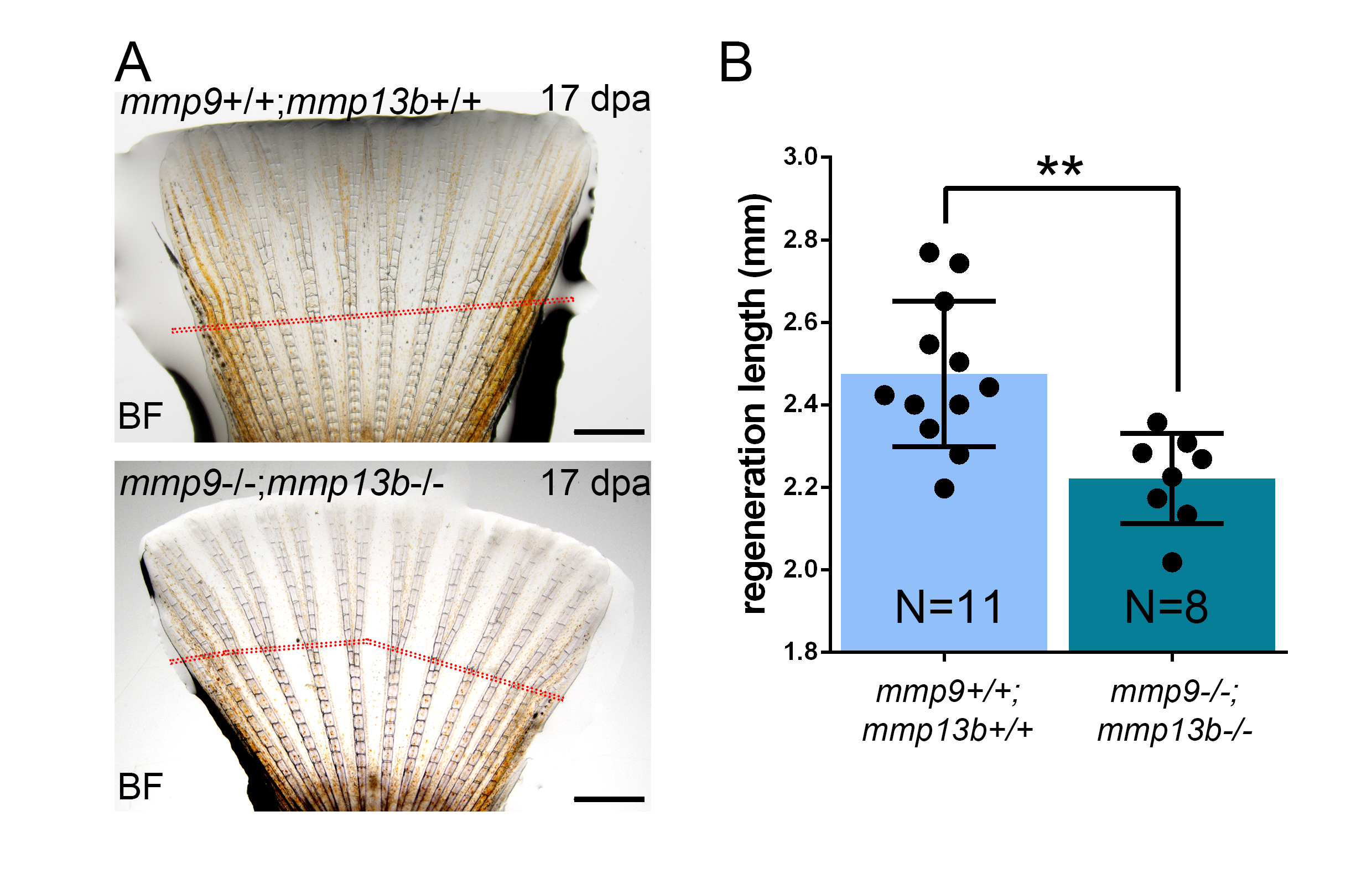

Supplement: Supplementary file 1 [file Image6.TIF]

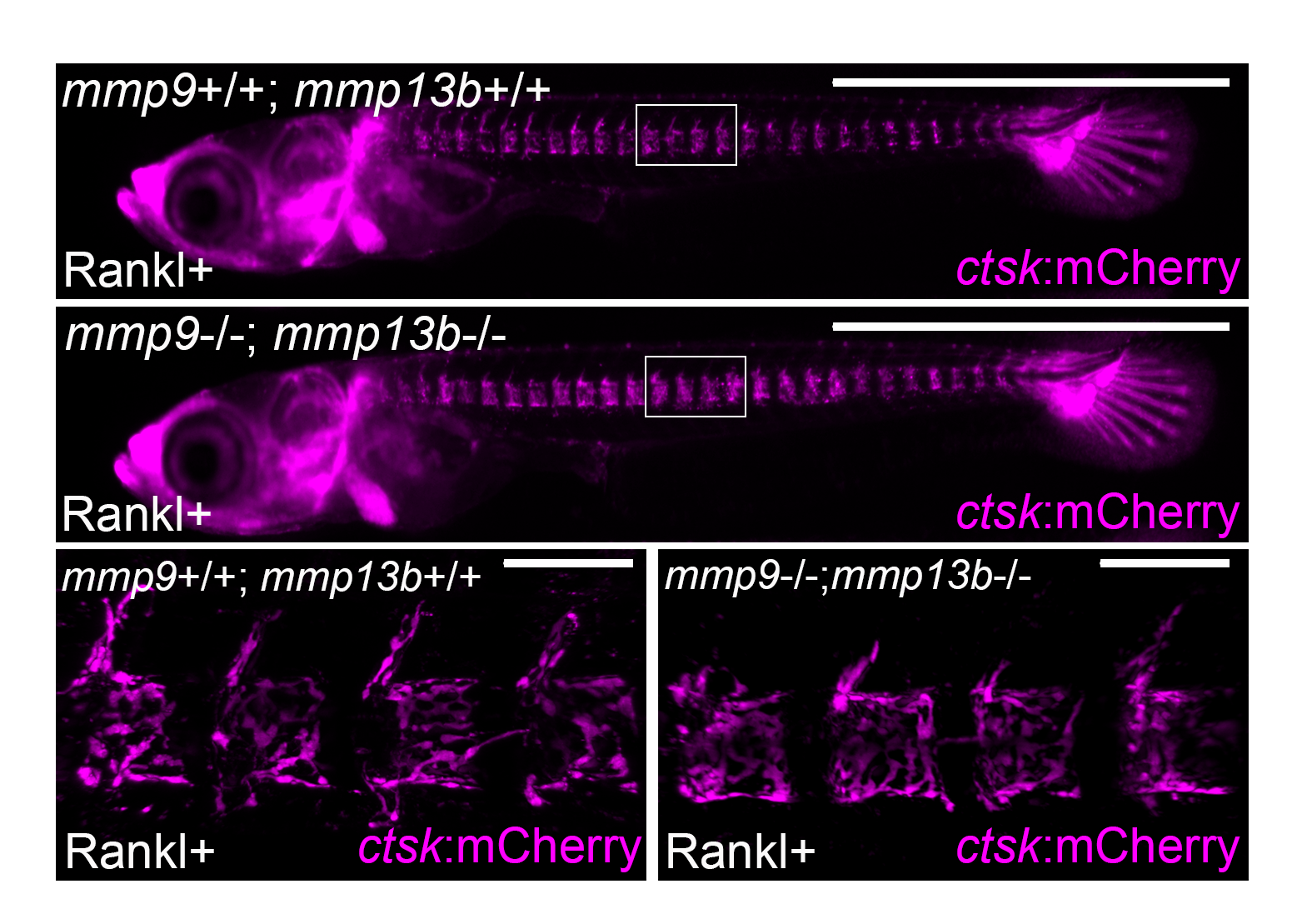

Supplement: Supplementary file 3 [file Image4.TIF]

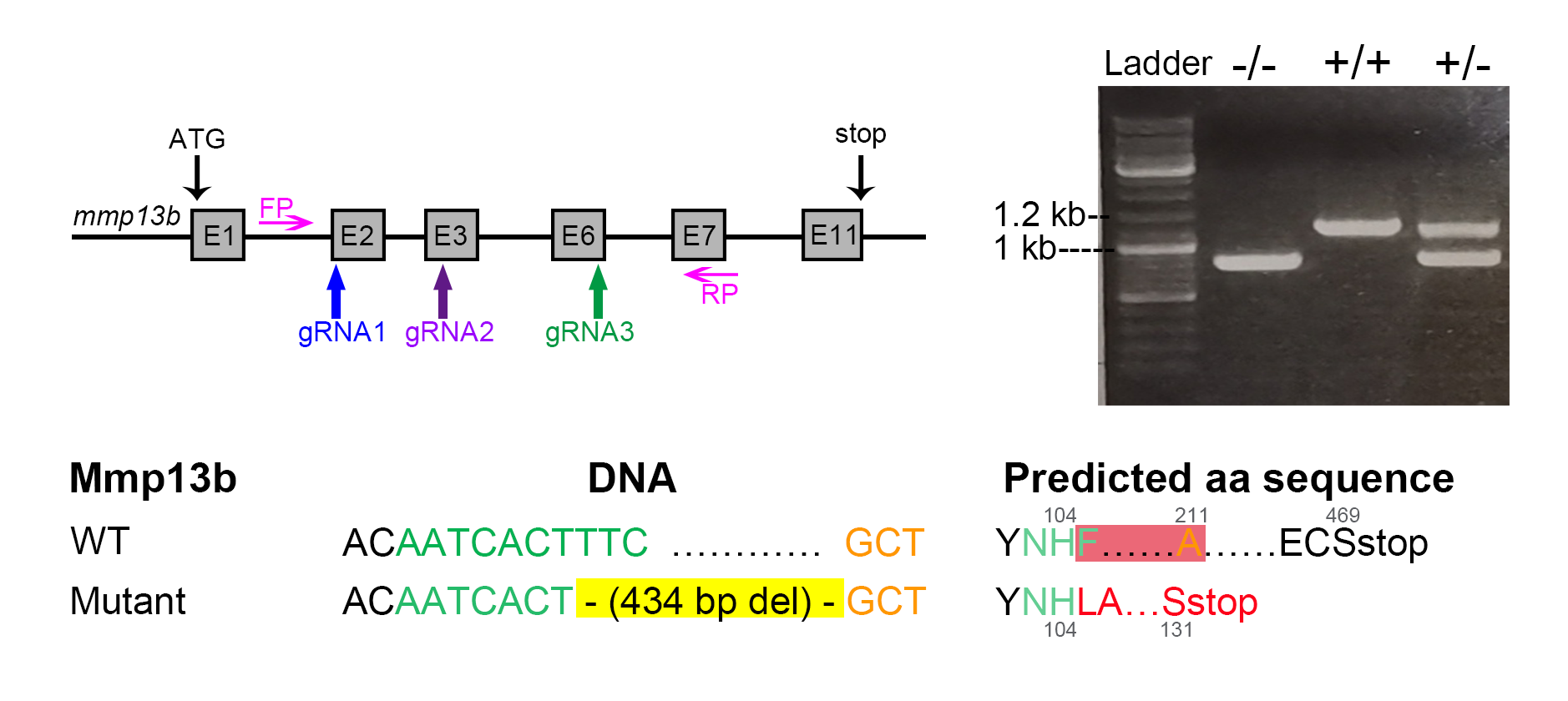

Supplement: Supplementary file 4 [file Image2.TIF]

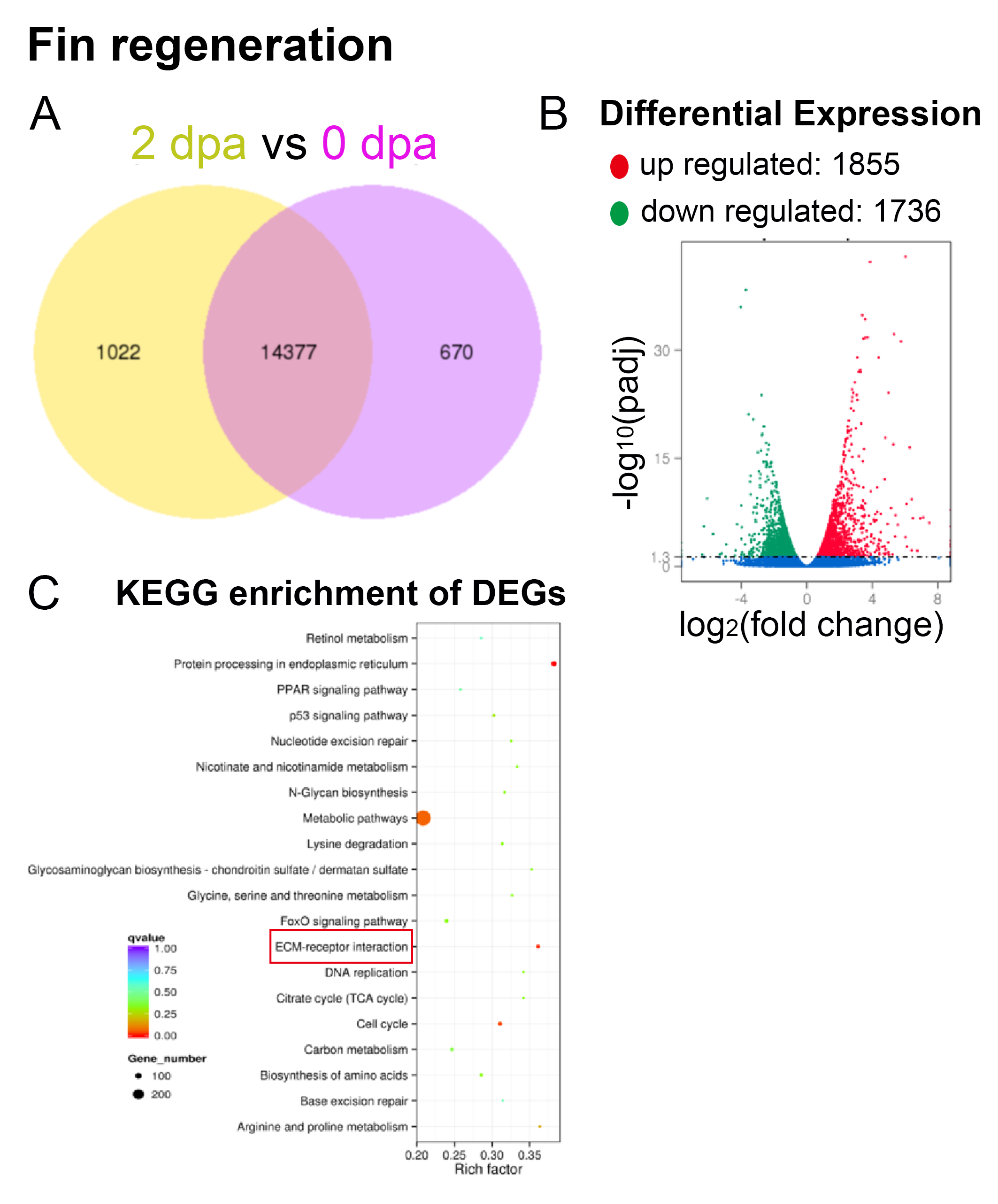

Supplement: Supplementary file 5 [file Image1.TIF]

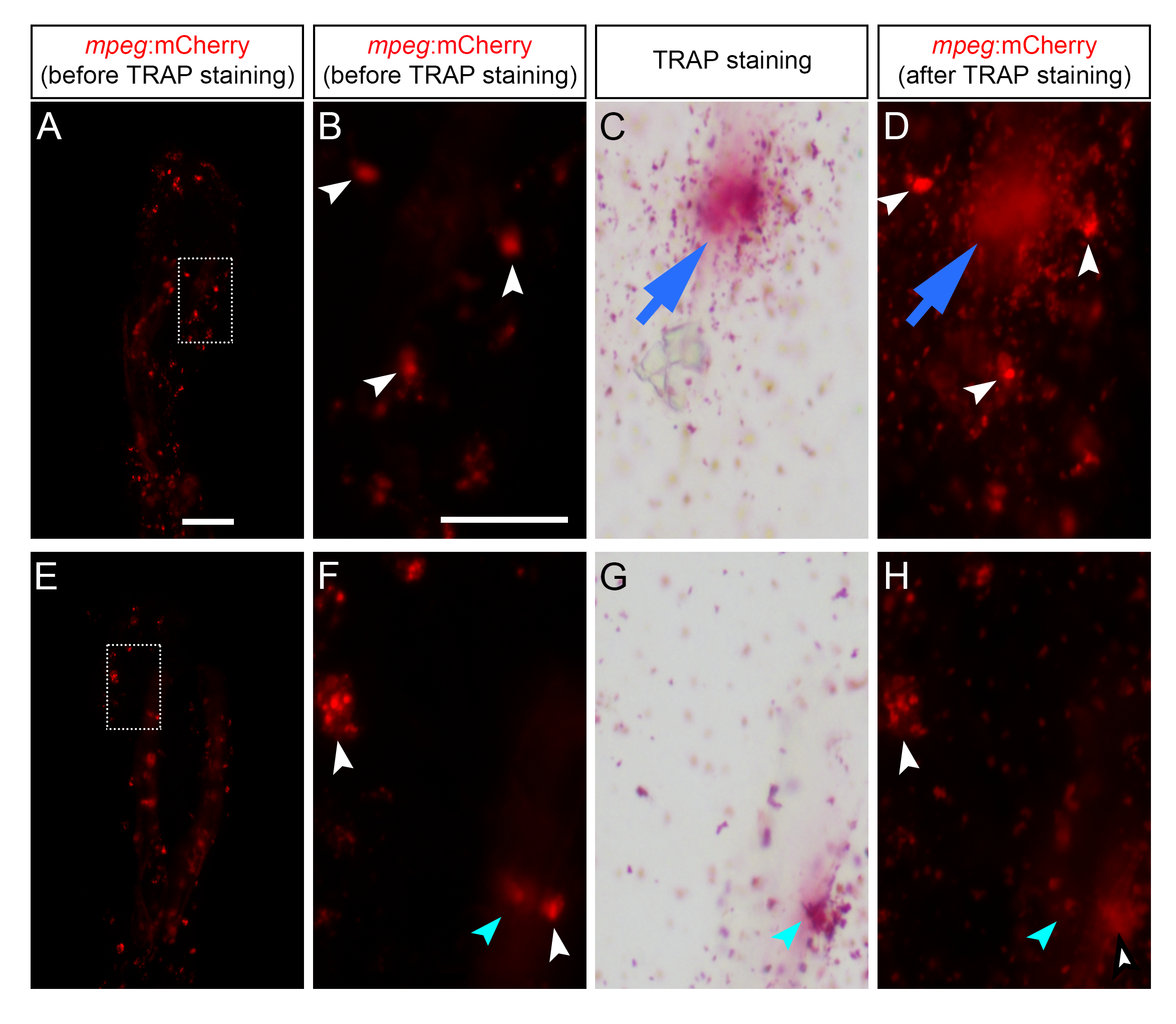

Supplement: Supplementary file 7 [file Image5.TIF]
