# Supplementary material for: Transcriptome Profiling of Osteoblasts in a Medaka (Oryzias latipes) Osteoporosis Model Identifies Mmp13b as Crucial for Osteoclast Activation
Source: Front Cell Dev Biol. 2022 Feb 21;10:775512. doi: 10.3389/fcell.2022.775512 (PMC8911226; doi:10.3389/fcell.2022.775512)
Supplement: Supplementary file 9 [file DataSheet1.docx]

**Supplementary Table S1. *mmp* transcripts regulated in medaka bone cells under osteoporotic conditions.**

| Gene Name | baseMean *col10a1* (10 dpf) | baseMean *osx*  (10 dpf) | baseMean *ctsk*  (12 dpf) | FC  *col10a1*  (10 dpf) | FC  *osx*  (10 dpf) | FC  *ctsk*  (12 dpf) |
| --- | --- | --- | --- | --- | --- | --- |
| *mmp13b* | 1052.85 | 772.16 | 27.6 | 59.95 | 7.17 | 1.64 |
| *mmp9* | 14765.11 | 9157.18 | 125.52 | 36.02 | 8 | 1.2 |
| *mmp14* | 4639.77 | 2886.14 | 450.32 | 3.51 | 1.98 | 1.5 |
| *mmp23b* | 226.72 | 237.48 | 89.83 | 2.58 | 1.51 | 1.19 |
| *mmp17b* | 8.44 | 22.63 | 12.23 | 2.5 | 2.12 | 1.57 |
| *mmp13 (collagenase)* | 48.34 | 111.12 | 78.15 | 2.28 | 1.47 | 7.09 |
| *mmp19 like* | 79.55 | 70.11 | 16.27 | 2.01 | 1.76 | 1.19 |
| *mmp19* | 278.98 | 198.94 | 384.82 | 1.82 | 1.03 | 1.09 |
| *mmp2* | 9014.54 | 13056.45 | 3850.28 | 1.74 | 1.22 | 1.5 |
| *mmp15b* | 92.44 | 83.01 | 37.34 | 1.49 | 1.03 | 1.45 |
| *mmp14a* | 3162.21 | 3344.94 | 1443.58 | 1.46 | 1.29 | 1.47 |
| *mmp16b* | 169.34 | 106.21 | 16.36 | 1.32 | 1.19 | 1.7 |
| *mmp15a* | 293.8 | 442.9 | 348.72 | 1.29 | 1.29 | 1.29 |
| *mmp28* | 24.14 | 11.32 | 10.33 | 1.21 | 1.14 | 1.69 |
| *mmp17a* | 151.47 | 150.39 | 63.15 | 1.17 | 1.28 | 1.89 |
| *mmp24* | 30.21 | 16.76 | 4.25 | 1.17 | 1.53 | 1.84 |
| *mmp20a* | 351.23 | 193.04 | 48.83 | 1.09 | 1.22 | 1.33 |
| *mmp21* | 2.96 | 5.31 | 2.28 | 1.04 | 7.89 | 1.43 |
| *mmp25* | 15.33 | 828.03 | 234.32 | 1.03 | 1.12 | 1.08 |
| *mmp17* | 1055.16 | 900.78 | 73.11 | 1.02 | 1.34 | 2 |
| *mmp16* | 231.34 | 272.47 | 200.15 | 1.01 | 1.3 | 1.16 |

**Supplementary Table S2. *mmp* transcripts regulated during fin regeneration.**

| Gene Name | read count  (0 dpa) | read count  (2 dpa) | fold change |
| --- | --- | --- | --- |
| *mmp16* | 72.46 | 553.54 | 7.64 |
| *mmp14* | 1201.85 | 7190.05 | 5.98 |
| *mmp13b* | 661.85 | 2947.97 | 4.45 |
| *mmp11b* | 279.62 | 813.08 | 2.91 |
| *mmp14a* | 2577.88 | 6877.89 | 2.67 |
| *mmp17b* | 0.86 | 1.84 | 2.14 |
| *mmp13*  *(collagenase)* | 2313.97 | 4203.08 | 1.82 |
| *mmp2* | 9068.87 | 16056.95 | 1.77 |
| *mmp9* | 28385.21 | 49822.61 | 1.76 |
| *mmp15a* | 408.56 | 700.14 | 1.71 |
| *mmp20a* | 226.08 | 345.92 | 1.53 |
| *mmp16b* | 10.15 | 15.25 | 1.5 |
| *mmp23b* | 115.73 | 158.53 | 1.37 |
| *mmp19* | 5077.49 | 6310.63 | 1.24 |
| *mmp25b* | 26.78 | 18.82 | 0.7 |
| *mmp17a* | 278.82 | 152.36 | 0.55 |
| *mmp28* | 389.64 | 163.66 | 0.42 |
| *mmp19 like* | 3347.83 | 1268.45 | 0.38 |
| *mmp24* | 0 | 1.74 | -- |

**Supplementary Table S3. Oligos used.**

| *mmp13b*_ISH_FP | GTCAAGCTCTGAACGTCTGGTC |
| --- | --- |
| *mmp13b*_ISH_RP | TCCATTCAAAGCCCACATTCGG |
| *β-actin*_qPCR_FP | GCCAACAGGGAGAAGATGAC |
| *β-actin*_qPCR_FP | CATCACCAGAGTCCATGACG |
| *mmp13b*_qPCR_FP | GCCTCACCTACAGGATCACAAAC |
| *mmp13b*_qPCR_RP | CTGCCGTGCTGCTGTAGATT |
| *mmp13b*_gRNA1 | AGACGATTACCTTTTAGCCGAGG |
| *mmp13b*_gRNA2 | CACCTGGCCTCCAAGGTCGAAGG |
| *mmp13b*_gRNA3 | CCTATTGGCACATGCCTACCCAC |
| *mmp13b*_FP | GCTGAGAGCATCTGGCAATCATT |
| *mmp13b*_RP | GGGCTGCTACTATGAACAGGTT |
| *mmp9_*qPCR_FP | CAGACCTGGCAGAGAGCTATCTAA |
| *mmp9_*qPCR_RP | GTCCAGTTGTCCTGTCTCGTCT |
| *traf6*_qPCR_FP | AGTCCATTCGTGATACAGGACAGC |
| *traf6*_qPCR_RP | CGGAATTTGGACAGCGAACAG |
| *ctsk*_qPCR_FP | ACAACACGAGCTATGTGGTCGT |
| *ctsk_*qPCR_RP | CCTCTTCCTCCACGGTGATGTAT |
| *itgb8*_qPCR_FP | AAGGAGGACTTTCTAGACAAGGTGGG |
| *itgb8_*qPCR_RP | GCGGAGACACTTGTGTCCTGGTGA |
| *rank*_qPCR_FP | AGATGCTAACGGAAACTGCGAG |
| *rank*_qPCR_RP | TCACTTTTGCCCAGAAGACACTG |
| *itga2.2_*qPCR_FP | TGG AAA CCT CTG ACC CAA ATC TGC |
| *itga2.2_*qPCR_RP | AAC GTC AGC TGT TTC TCC AGG |
| *TRAP*_qPCR_FP | CGCTTCCAGGAAACCTTCGAG |
| *TRAP*_qPCR_RP | CTCTGGGAGTACTCAATCTGGG |
| *col6a1*_qPCR_FP | AAT CTC ACA GCC AAG ATC TGC C |
| *col6a1*_qPCR_RP | TGG CTG AGC TGT CCA TCA TG |
| *col6a3*_qPCR_FP | AGACGGAGCAGGTGGAGATTC |
| *col6a3*_qPCR_RP | ACCATTTCAGGGCGAACTCTG |
| *il10*_qPCR_FP | TCAGGACTGATGTCACCCAATG |
| *il10*_qPCR_RP | TCCCATGGCCTTGAAGAGAC |
| *mmp9*_FP | GTTCATGGAGTGTTCCCCTCA |
| *mmp9*_RP | CCATCGAAGAGGCGGGTAAA |
| *mmp9*_gRNA1 | ATGTTCTTTATGACATCTCCAGG |
| *mmp9*_gRNA2 | CCAGTTGTCCTGTCTCGTCTAGG |
| *mmp9*_gRNA3 | ATCATGGTGATGTTACGTATAGG |
| mmp9_cDNA1_FP | GAGCCCAGGATCAAAACAAAGA |
| mmp9_cDNA1_RP | GCATCTCCCTGTATGCCCTC |
| mmp9_cDNA2_FP | TAGGGATGGCTACCGTTGGT |
| mmp9_cDNA2_RP | GGCAAGCCAAGCTTCTCAAT |

**Supplementary Table S4. Numbers of histological sections used for experiments in Figures 3 and 6.**

| Numbers of sections used | | | | |
| --- | --- | --- | --- | --- |
|  | | *mmp13b* +/+ | *mmp13b* -/- |  |
| Figure 3 | Picro-Sirius red staining | 27 | 34 | Rankl- |
|  |  | 27 | 35 | Rankl+ |
|  | Collagen 1 IHC on larvae | 27 | 28 | Rankl- |
|  |  | 20 | 20 | Rankl+ |
| Figure 5 | Collagen *in situ* zymography | 10 | -- | Rankl-,  *osx*:mCherry |
|  |  | 24 | -- | Rankl+,  *osx*:mCherry |
|  |  | 22 | -- | Rankl+,  *ctsk*:mCherry |
|  |  | -- | 57 | Rankl+,  *ctsk*:mCherry |
| Figure 7 | Collagen1 IHC 0 dpa fins | 17 | 18 |  |
|  | Collagen1 IHC 2 dpa fins | 15 | 17 |  |
|  | Collagen 2 IHC 0 dpa fins | 17 | 18 |  |
|  | Collagen 2 IHC 2 dpa fins | 16 | 17 |  |
|  | Fibronectin IHC 0 dpa fins | 19 | 25 |  |
|  | Fibronectin IHC 2 dpa fins | 18 | 19 |  |
|  | Tenascin-C IHC 0 dpa fins | 21 | 27 |  |
|  | Tenascin-C IHC 2 dpa fins | 32 | 31 |  |
|  | *col10a1* ISH 6 dpa fins | 30 | 25 |  |
|  | TRAP staining 2 dpa fins | 26 | 17 |  |
